# SUMOylation inhibition enhances dexamethasone sensitivity in multiple myeloma

**DOI:** 10.1186/s13046-021-02226-9

**Published:** 2022-01-04

**Authors:** Li Du, Wei Liu, Grace Aldana-Masangkay, Alex Pozhitkov, Flavia Pichiorri, Yuan Chen, Steven T. Rosen

**Affiliations:** 1 Toni Stephenson Lymphoma Center, Beckman Research Institute of City of Hope, Duarte, CA USA; 2 Judy and Bernard Briskin Center for Multiple Myeloma Research, Beckman Research Institute of City of Hope, Duarte, CA USA; 3 Department of Hematology and Stem Cell Transplant, Beckman Research Institute of City of Hope, Duarte, CA USA; 4 Department of Hematology, Xiangya Hospital, Central South University, Changsha, China; 5 Department of Molecular Medicine, Beckman Research Institute of City of Hope, Duarte, CA USA; 6 Present address: Fulgent Genetics, City, Temple City, CA 91780 USA; 7 Department of Informatics, City of Hope National Medical Center, Duarte, CA USA; 8 Department of Surgery and Moores Cancer Center, UC San Diego Health, San Diego, CA USA; 9 City of Hope Comprehensive Cancer Center, City of Hope National Medical Center, Duarte, CA USA

**Keywords:** Multiple myeloma, SUMOylation, Dexamethasone, microRNA

## Abstract

**Background:**

Multiple myeloma (MM) is an incurable plasma cell malignancy. Although Dexamethasone (Dex) is the most widely used therapeutic drug in MM treatment, patients develop Dex resistance leading to progressive disease, demanding an urgent need to investigate the mechanisms driving Dex resistance and develop new reagents to address this problem. We propose SUMOylation as a potential mechanism regulating Dex resistance and SUMOylation inhibition can enhance Dex sensitivity in MM.

**Methods:**

Using MM cell lines and primary MM samples from relapsing MM patients, we evaluated the effects of knockdown of SUMO E1 (SAE2) or using TAK-981, a novel and specific SUMO E1 inhibitor, on Dex sensitivity. Xenograft mouse models were generated to determine the in vivo anti-MM effects of TAK-981 as a single agent and in combination with Dex. miRNA-seq, RNA-seq and GSEA analysis were utilized for evaluating key factors mediating Dex resistance. Chromatin immunoprecipitation (ChIP) assay was performed to determine the binding occupancy of c-Myc on promoter region of miRs.

**Results:**

We observed a significant negative correlation between SUMO E1 (SAE2) expression and Dex sensitivity in primary MM samples. Knockdown of SAE2 or using TAK-981 significantly enhances myeloma sensitivity to Dex in MM cell lines. Moreover, the enhanced anti-MM activity by TAK-981 and Dex combination has been validated using primary relapsing MM patient samples and xenograft mouse models. SUMOylation inhibition increased glucocorticoid receptor (GR) expression via downregulation miR-130b. Using RNA and microRNA sequencing, we identified miR-551b and miR-25 as important miRs mediating Dex resistance in MM. Overexpression of miR-551b and miR-25 caused resistance to Dex, however, knockdown of miR-551b and miR-25 significantly enhanced Dex sensitivity in MM. SAE2 knockdown or TAK-981 treatment downregulated the expression of miR-551b and miR-25, leading to induction of miR targets ZFP36, ULK1 and p27, resulting in apoptosis and autophagy. We demonstrated c-Myc as a major transcriptional activator of miR-130b, miR-551b and miR-25 and SUMOylation inhibition downregulates these miRs level by decreasing c-Myc level.

**Conclusion:**

Our study proves SUMOylation plays a crucial role in Dex resistance in MM and SUMOylation inhibition appears to be an attractive strategy to advance to the clinic for MM patients.

**Supplementary Information:**

The online version contains supplementary material available at 10.1186/s13046-021-02226-9.

## Introduction

Multiple myeloma (MM), the second most common hematologic cancer is an incurable plasma cell malignancy. Patients with MM accumulate monoclonal immunoglobulin-producing plasma cells within their bone marrow. Despite several new drugs that have improved the survival of myeloma patients in the past decade, patients typically develop relapsed and/or refractory MM and long-term disease-free survival remains low [1]. Dexamethasone (Dex), a synthetic glucocorticoid (GC), is most widely used in MM combination regimens. Intrinsic or acquired Dex resistance is broadly associated with poor prognosis in MM [2, 3]. Although the mechanisms of Dex resistance, including defective of glucocorticoid receptor (GR) expression, dysregulation of apoptosis-mediating proteins or small non-coding RNAs (miRNAs, miRs) have been described, the underlying regulatory mechanisms are not fully elucidated [2, 4].

SUMOylation is a post-translational modification characterized by covalent attachment of small ubiquitin-like modifier (SUMO) proteins to a lysine (Lys) residue on target proteins [5–7]. It is carried out via an enzymatic cascade involving the sequential action of an activating enzyme E1 (a heterodimer of SAE1 and SAE2), a conjugating enzyme E2 (UBC9), and a ligating enzyme E3 (one of ~ 10). SUMOylation enzymes are expressed at higher levels in cancer cells than in normal cells. Their elevated expression is required for tumor progression, cancer metastasis, and cancer stem cell maintenance and self-renewal. The expression of SUMOylation enzymes is usually associated with poor survival in various human cancers, including MM, colorectal (CRC), and breast cancers [8–11]. SUMOylation enzymes showed associated with poor survival in MM, suggesting SUMOylation might be related with therapy resistance. Because Dex is most widely used in MM combination therapy, we investigated if SUMOylation could be a potential mechanism of Dex resistance in MM.

In this study, we found that the expression level of SUMO E1 (SAE2) was inversely correlated with Dex sensitivity of primary MM samples. SUMOylation inhibition by decreasing SUMO E1 level via shRNA knockdown or inhibiting SUMO E1 activity by TAK-981 greatly enhanced MM sensitivity to Dex treatment. TAK-981, a novel selective SUMO E1 inhibitor, is currently in clinical trials for lymphoma and solid tumors. Our study demonstrated that TAK-981 was effective against primary relapsing MM samples and in mouse xenograft models. Furthermore, the combination of TAK-981 with Dex showed potent synergistic anti-MM effects ex vivo and in vivo. Using microRNA sequencing and Chromatin immunoprecipitation (ChIP) assay, we investigated a mechanism by which SUMOylation inhibition enhanced MM sensitivity to Dex. Our data suggest that inhibiting the SUMO E1 enzyme is a novel therapeutic strategy to overcome Dex resistance and prove TAK-981 as a potent clinical drug for enhancing current therapeutics in MM patients.

## Methods

### Reagents

TAK-981 was purchased from ChemieTek. Dexamethasone was purchased from Cayman Chemistry company. Dexamethasone-water soluble was purchased from Sigma (D2915) for animal experiment.

### Multiple myeloma cell lines and primary samples

Primary MM cells were isolated from bone marrow aspirates of MM patients, using Ficoll-Hypaque density gradient sedimentation followed by CD138 microbeads separation (Miltenyi Biotec), with informed patient consent and the Research Ethics Board approval at City of Hope (IRB 15150). Human myeloma cell lines MM1S, MM1R, H929 and RPMI8226 (obtained from ATCC) and primary CD138+ MM cells were cultured in RPMI1640 medium (Corning) with 10% heat-inactivated FCS (Omega Scientific, Inc.), 2 mmol/L l-glutamine, and 1% antibiotic-antimycotic (Life Technologies). Mycoplasma was routinely tested using MycoAlert Mycoplasma Detection Kit (LT07–318, Lonza) or Mycoplasma PCR detection kit (G238, Abm).

RPMI-8226 cells stably expressing Tet-R were used for lentivirus transduction within one to two passages after blasticidin selection. For lentivirus generation, the envelope plasmid pCMV-VSVG and the packaging plasmid pCMV-dR8.2-dvpr were obtained from Addgene (8454 and 8455, provided by Dr. Bob Weinberg). Inducible SAE2 shRNAs were purchased from GE Dharmacon (V2THS_254939 and V2THS_68114). Inducible human Myc shRNA was also purchased from GE Dharmacon (V2THS_152051). 293 T producer cells were transfected with these vectors, and supernatant containing lentiviral particles was harvested 24–48 h after transfection. Then the cells were transduced with two different Tet-On shSAE2 or shMyc lentiviruses and the stably transduced cells were selected with puromycin (5 μg/ml) 2 days after viral transduction. For doxycycline (DOX)-induced SAE2 or c-Myc knockdown, 5 μg/ml DOX was added to cells for 3 days to induce knockdown.

### Cell viability assay and drug-synergy calculations

Cells (0.5-2 × 10^4^ cells/well) were seeded in 96-well plates and treated with the indicated reagents for 48 h. Cell viability assays were performed using CellTiter-Glo Luminescent Cell Viability (G7572, Promega) according to manufacturer’s instruction. Median inhibitor concentration (IC_50_) was determined using GraphpadPrism 8.0. Combination indices (CIs) were calculated using CalcuSyn software (Biosoft, Cambridge, UK), and values < 1.0 were considered to indicate synergy.

### Flow cytometry-based apoptosis assay

Cell apoptosis was measured after Annexin V FITC and 7-AAD staining using a BD Fortessa LSR II and FlowJo Version V10.6.2.

### RNA extraction, reverse transcription and quantitative real-time PCR (qRT-PCR)

Total cellular RNA was extracted using miRNeasy Mini Kit (Qiagen). Total RNA (2 μg) was reverse-transcribed using an Omniscript RT Kit (Qiagen) and oligo dT primer. miRNA cDNA was synthesized from total RNA using TaqMan miRNA Reverse Transcription Kit and 5X miRNA specific RT primer (Life Technology) according to manufacturer’s protocol. Real-time qPCR of gene expression was performed using the SYBR-Green Master Mix (Applied Biosystems and the results were normalized to GAPDH. For miRNA cDNA real-time PCR, TaqMan Universal PCR master mix (no UNG) (Life Technology) and Taqman Small RNA Assay mix (Life Technology) were used. All quantitative PCR reactions were performed using ViiA 7 real-time PCR system (Applied Biosystem). Relative expression was calculated using the comparative Ct method normalized to RNU6B. Primers were listed in the supplementary file*.*

### Western blot

Cells were harvested and lysed in Laemmli sample buffer (5% SDS, 25% glycerol, 150 mmol/L Tris-HCl pH 6.8, 0.01% bromophenol blue). After protein concentration was measured using BCA protein assay, 0.7 mol/L β-mercaptoethanol was added and protein samples were boiled for 10 min. Samples were separated by SDS-PAGE, and protein was transferred onto a polyvinylidene fluoride membrane (Immobilon-P membrane, Millipore). Following antibodies were used: SUMO-2,3 (M114–3, MBL), c-Myc (ab32072, Abcam), cleaved PARP (#5625S, Cell Signaling Technology), GR (#12041S, Cell Signaling Technology), TTP(A-8) (sc-374,305, Santa Cruz Biotechnology), RRM2 (#65939S, Cell Signaling Technology), ULK1 (#8054S, Cell Signaling Technology), Cyclin D1(#55506S, Cell Signaling Technology), E2F1(#3742S, Cell Signaling Technology), LC3B(#3868S, Cell Signaling Technology), p27 (3686S, Cell Signaling Technology), GAPDH (sc-20,357, Santa Cruz Biotechnology). Western blot results were visualized using an Odyssey detection system (Licor). Relative protein level was quantified by Image J and normalized to GAPDH.

### Ribo-zero RNA-seq, small RNA-seq and gene set enrichment analysis (GSEA)

MM1S and MM1R cell lines treated with Vehicle (Veh), TAK-981 at 0.1 μM (TAK), Dexamethasone 1 μM (Dex) or both TAK-981 and Dex (combo, Com) for 48 h. Total RNA was purified using miRNeasy RNA isolation kit (Qiagen) then Ribo-Zero RNA-seq and small RNA-seq were performed. Global mRNA and miRNA expression profiles of duplicated RNA samples were performed and analyzed by the City of Hope Integrative Genomics Core Facility. Gene set enrichment analysis (GSEA) was performed with gene sets downloaded from the Broad Institute’s MSigDB website (www.broad.mit.edu/gsea/) and data analysis was performed using web-based software. A FDR-adjusted *P* value less than 0.05 and FDR-adjusted *q*-value less than 0.25 were used as cutoffs to determine significance. All RNA-seq and miRNA-seq data have been deposited to the GEO database (GSE 163682).

### siRNA transfection

siRNA transfection was performed using Lipofectamine RNAiMAX (Life Technology) according to the manufacturer’s instructions. Cells were harvested at indicated time points for further analysis. Hsa-miR-25 mimic (Sigma HMI0413), hsa-miR-25 inhibitor (Sigma HSTUD0413), hsa-miR-551b mimic (Sigma HMI0771), hsa-miR-551b inhibitor (Sigma HSTUD0771), siRNA universal negative control (Sigma SIC001) were purchased from Sigma. SiRNAs against SAE2, UBC9 and c-Myc were obtained from GE Dharmacon SMARTpool.

### Chromatin immunoprecipitation assay

To detect the binding occupancy of c-Myc on the human microRNA miR-130b, miR-551b and miR-25 promoter, chromatin immunoprecipitation (ChIP) analysis was conducted using SimpleChIP Enzymatic Chromatin IP kit (Magnetic Beads) (#9003, Cell Signaling Technology). A total of 2 × 10^7^ MM1S or MM1R cells were incubated in culture medium containing 1% formaldehyde for 10 min at room temperature, after which, the cross-linking reaction was quenched with addition of glycine to a final concentration of 0.125 mol/L. Cells were washed with cold PBS and harvested, followed by sonication to produce chromatin of primarily mononucleosome size. Fragmented chromatin was then incubated with c-Myc antibody at 4 °C overnight. Protein–DNA complexes were recovered using protein G dynabeads, washed, and eluted with elution buffer. Crosslinks were reversed at 65 °C in 0.25 mol/L NaCl overnight; then, the DNA was digested with proteinase K for 2 h at 50 °C. The immunoprecipitated DNAs were subsequently isolated and used for qPCR.

### Tumor xenograft mouse model

All experiments using mice were approved by the Beckman Research Institute Animal Care and Use Committee (IACUC #10026) and complied with all relevant federal guidelines and institutional policies. MM1S cells with stably expression of GFP-firefly luciferase (fflucGFPMM.1S) (Gift from Dr. Xiuli Wang, City of Hope) was intravenously (i.v.) injected to 6 ~ 10-week old NOD/ScidIL2RrC mice via tail vein (2 × 10^6^ cells/mouse) via tail vein. After 10 days, tumor xenografts were confirmed by bioluminescence imaging and mice were randomized into two groups (6 mice for each group) and treated with Vehicle control or TAK-981 (7.5 mg/kg, intraperitoneally (i.p.) twice per week, BIW) for 2 weeks. Tumor growth was monitored by quantification of bioluminescence mice whole body imaging using a Xenogen IVIS 100 series system (Xenogen, Alameda, CA). For MM1R cell xenograft model, MM1R cells (4 × 10^6^/100 μl with Matrigel) were subcutaneously (s.c.) inoculated into the mouse hind flank. When tumors were palpable, mice were randomly assigned to 4 groups for treatments—Vehicle control (*n* = 8), Dex group (3 mg/kg, i.p. BIW) (*n* = 4), TAK-981 (10 mg/kg, s.c. BIW) (*n* = 8), or combination group (Dex 3 mg/kg i.p. and TAK-981 10 mg/kg s.c. BIW) (*n* = 8). Tumor volume was measured with calipers until tumor diameter reached 1.80 cm. Then, mice were euthanized, and tumor tissues were harvested for tissue RNA extraction and immunohistology analysis.

### Immunohistochemistry (IHC)

For IHC staining of cleaved-PARP and CD138, tumor tissues were fixed in 4% paraformaldehyde, embedded in paraffin and IHC was performed by City of Hope Solid Tumor Pathology Core facility according to standard procedures.

### Protein degradation assay

c-Myc protein stability was measured on treatment with protein synthesis inhibitor cycloheximide (CHX). Cells treated with 100 μg ml^− 1^ CHX (#2112, Cell Signaling Technology) were collected at different time points and cell lysate was used for western blot to determine the protein level at different CHX treatment time. Western blot results were quantified by the ImageJ Software (NIH).

### Ni-NTA assay

Nickel-charged affinity (NTA) magnetic agarose beads (Thermo Fisher #78605) were used to perform His pull down in cells with overexpression of His-tagged SUMOs. The plasmid c-myc-PT3EF1a was a gift from Xin Chen (Addgene plasmid # 92046; http://n2t.net/addgene:92046; RRID:Addgene_92,046). Plasmid hSUMO1-HIS was a gift from Lea Sistonen (Addgene plasmid # 118361; http://n2t.net/addgene:118361; RRID:Addgene_118,361) and pcDNA3-6XHis-human SUMO 2 was a gift from Martine Roussel (Addgene plasmid # 133771; http://n2t.net/addgene:133771; RRID:Addgene_133,771) [12–14]. 293 T cells were transfected with His-tagged SUMO-1, His-tagged SUMO-2 and untagged c-Myc expression plasmids. Cells were treated with 0.1 μM TAK-981(TAK) or Vehicle (Veh) for 8 h. Cell lysates were incubated with Ni-NTA beads to pull down all His-tagged SUMO-1 and SUMO-2, followed by Western blot.

### Statistical analyses

No animals or samples were excluded from analysis. For all experiments, *P* values were derived using a two-tailed Student t test (comparing 2 conditions) or ANOVA (comparing multiple conditions) using GraphPad Prism 8. Estimated variation is indicated as means and error bars depict standard deviation (SD) in each figure. For all graphs, *, *P* < 0.05; **, *P* < 0.01; and ***, *P* < 0.001.

## Results

### Expression of SUMO E1 is upregulated in multiple myeloma and associated with poor prognosis

mRNA profiling of myeloma patient cohort GSE5900 showed that SUMO E1 (Ubiquitin Like Modifier Activating Enzyme 2(UBA2)/SUMO activating enzyme 2 (SAE2)) gene expression was significantly upregulated at the premalignant myeloma stage (MGUS) and in the asymptomatic myeloma phase (smoldering MM) compared to healthy plasma cells (Supplemental Fig. S1A). Moreover, higher SUMO E1 (UBA2) gene expression in MM plasma cells was associated with significantly shorter survival in both GSE2658 (*n* = 559) and CoMMpass (clinical trial NCT01454297, *n* = 764) database (Supplemental Fig. S1B&C) [15, 16]. These data suggest that SAE2 might be associated with therapy resistance in MM.

### SUMOylation inhibition enhances Dex anti-MM activity in primary patient samples and cell lines

**Fig. 1:**
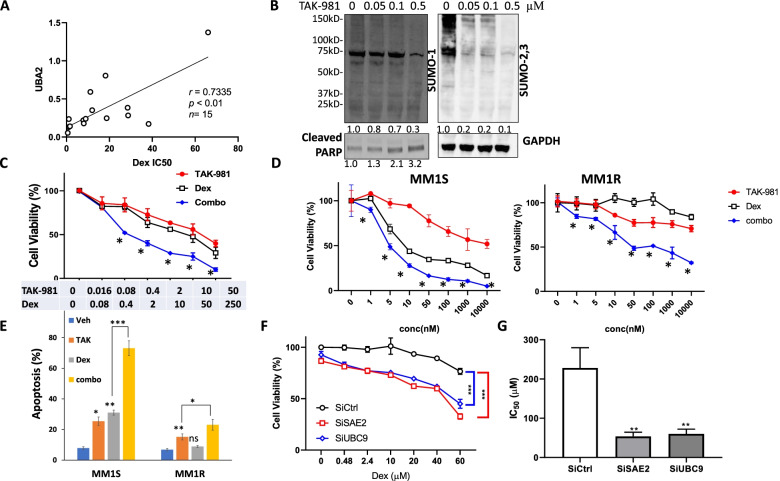
SUMOylation inhibition synergizes with Dex in decreasing cell viability in primary multiple myeloma cells and MM cell lines. **A** SAE2 expression is associated with Dex sensitivity in primary MM cells. SAE2 (UBA2) mRNA expression was assessed by q-PCR and presented as relative level normalized to GAPDH. IC_50_ values were calculated by GraphPad Prism 8. Pearson correlation analysis showed a significant positive correlation between SAE2 mRNA and IC_50_ for Dex in 15 primary MM. **B** TAK-981 inhibits global SUMOylation and induces cleaved PARP in MM1S cells. Western blot presents SUMO-1, SUMO-2,3-modified protein in MM1S cells after exposure to TAK-981 at the indicated concentrations for 16 h. GAPDH serves as loading control. Relative protein level was quantified and labeled below each blot. **C** Cell viability assay showing 1 of 6 primary CD138+ cells from bone marrow aspirates of MM patients treated with TAK-981 or Dex or both (combo) with indicated concentration. Cell viability was assessed by Cell-Titer-Glo after 48 h of treatment. *Combination Index (CI) < 1 determined by CompuSym. **D** TAK-981 synergizes with Dex cytotoxicity in sensitive line (MM1S) and resistant line (MM1R). MM1S and MM1R cells were treated with indicated concentration of TAK-981 or Dex or both (combo) and cell viability was determined by Cell-Titer-Glo post-48 h treatment. *Combination Index (CI) < 1 determined by CompuSym. **E** TAK-981 enhances cytotoxicity of Dex in sensitive and resistant MM. MM1S and MM1R cells were treated with Vehicle (Veh), 0.1 μM TAK-981 (TAK), 1 μM Dex (Dex), or 0.1 μM TAK-981 with 1 μM Dex (combo). Apoptosis was measured by flow cytometry using Annexin V/PI staining. **F** Knockdown of SAE2 and UBC9 enhances MM1R Dex sensitivity. MM1R cells were transfected with siRNA targeting SAE2 (SiSAE2) or UBC9 (SiUBC9), or non-targeting control (SiCtrl) for 48 h then treated with different concentration of Dex for 24 h. Cell viability was measured and normalized to untreated control cells. **G** IC_50_ values of Dex in MM1R cells calculated from data in **F**. Data were analyzed using unpaired Student *t* tests: Data presented as mean ± SD. ns, not significant; *, *p* < 0.05; **, *p* < 0.01; ***, *p* < 0.001

Considering Dex is the most widely used therapeutic drug in MM treatment, we started with investigation of SAE2/UBA2 expression and sensitivity to Dex treatment. We correlate expression of UBA2 mRNA levels with the sensitivity of Dex in primary MM cells isolated from relapsing MM patients. We treated CD138+ MM cells from bone marrow aspirates of 15 patients with different Dex concentrations and assayed the cell viability post 48 h treatment. The half maximal inhibitory concentrations (IC_50_s) were calculated and compared with individual UBA2 mRNA expression across all primary samples by Pearson correlation coefficients (Fig. 1A). We observed that the expression level of UBA2 showed a significant positive correlation with Dex IC_50_s, indicating UBA2 expression is associated with Dex resistance in MM cells.

TAK-981, a novel, selective small molecule inhibitor of SUMO E1 enzyme, is currently in Phase 1 trials in adult patients with metastatic solid tumors and lymphomas [17]. We treated MM1S cells with TAK-981, detected SUMOylated proteins using anti-SUMO-1 and anti-SUMO-2,3 antibodies (Fig. 1B). TAK-981 inhibited global SUMOylation in a dose-dependent manner along with induction of apoptosis marker cleaved PARP. We then tested the impact of TAK-981 as a single agent and in combination with Dex in MM. In primary MM cells isolated from relapsing myeloma patients, combination treatment of TAK-981 with Dex resulted in significantly enhanced killing compared to the single agents alone (*n* = 6) (Fig. 1C). Consistent synergistic effects were observed in all 6 tested MM patient samples and the combination index was calculated (Supplemental Table S1).

MM1R cell line is resistant to Dex and its parental sensitive counterpart is MM1S. We conducted cytotoxicity assay in MM1S and MM1R cell lines, TAK-981 synergized with Dex in both cell lines (Fig. 1D). Dex showed limited effects on inducing apoptosis or inhibiting cell proliferation in MM1R cells. In contrast, TAK-981 induced cell apoptosis and decreased ell viability in both cell lines and the effects were further enhanced in combination with Dex (Fig. 1E). The synergistic effects of combination TAK-981 and Dex were observed in other MM cell lines H929 and RPMI8226 (Supplemental Fig. S2A).

To verify the effect of TAK-981 is specific through regulating SUMOylation, we conducted knockdown experiments. Transient (siRNA) knockdown of SUMO E1, SAE2 and SUMO E2, UBC9 significantly increased Dex sensitivity in MM1R cells (Fig. 1F, Supplemental Fig. S2B-C). Furthermore, we established stable cell lines that can be induced by doxycycline (dox) to express shRNA targeting SAE2 in the MM cell line RPMI8226. Two individual shRNAs (shSAE2#1 and shSAE2#5) were used to rule out nonspecific shRNA effects. Cells were treated with Dox for 3 days to achieve knockdown of SAE2 expression in both stable lines (Supplemental Fig. S3D), and then cell viability was measured. SAE2 knockdown significantly reduced MM cell viability (Supplemental Fig. S3E). More importantly, cells with stably knockdown of SAE2 showed a significant response to Dex treatment with decrease of IC_50_ values in both stable lines (Supplemental Fig. S3F). These data indicated SUMOylation is associated with Dex resistance and SUMOylation inhibition enhances Dex sensitivity.

### TAK-981 shows anti-MM activity in vivo

**Fig. 2:**
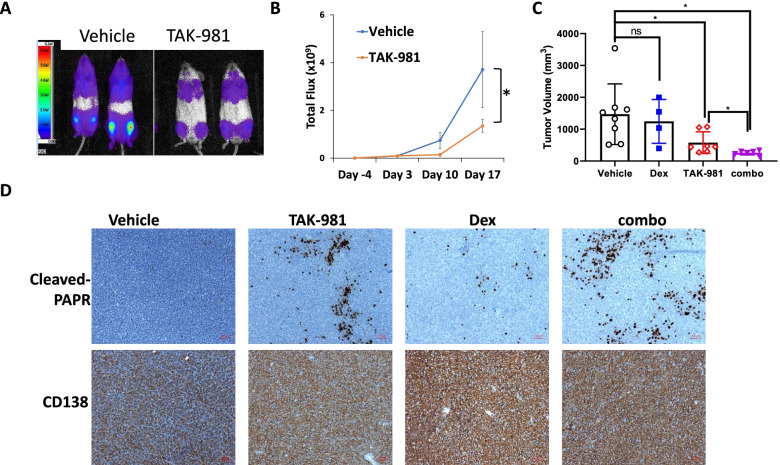
TAK-981 inhibits MM tumor growth and synergizes with Dex effect in vivo*.* (A) TAK-981 treatment suppresses tumor growth in MM1S-ffGFP xenograft NSG mice. MM1S-ffGFP cells were intravenously (iv) injected to NSG mice via tail vein (1.5 × 10^6^ cells/mouse). After bioluminescence imaging to confirm tumor engraft, mice were randomly assigned to two groups (*n* = 6) and treated with TAK-981 at 7.5 mg/kg or vehicle twice a week. Tumor growth was monitored by weekly imaging and quantified by Aura software. **A** Bioluminescence image representative 2 out of 6 mice from each group on Day 10 of treatment. **B** Tumor growth curve determined by the bioluminescence signal was measured as total photon flux normalized for exposure time and surface area and expressed in units of photons (p) per second per cm2 per steradian (sr). **C** TAK-981 treatment suppresses tumor growth in MM1R xenograft NSG mice model with synergistic effect in combination with Dex. NSG mice were xenografted by subcutaneously injection of MM1R cells (4 × 10^6^ cells /mouse). Mice were treated with either vehicle, or Dex (3 mg/kg), TAK-981 (10 mg/kg) or combination of both Dex and TAK-981(combo). All agents were treated twice weekly (BIW). Tumor growth was evaluated weekly by caliper measurement and represented as tumor volume (millimeters cubed). Comparison of tumor volume on end point was plotted. **D** IHC staining of apoptosis marker cleaved PARP and myeloma marker CD138 expression in xenograft tumor tissues. Red bar represents 50 μm. Data were analyzed using unpaired Student *t* tests: Data presented as mean ± SD. ns, not significant; *, *p* < 0.05

**Fig. 3:**
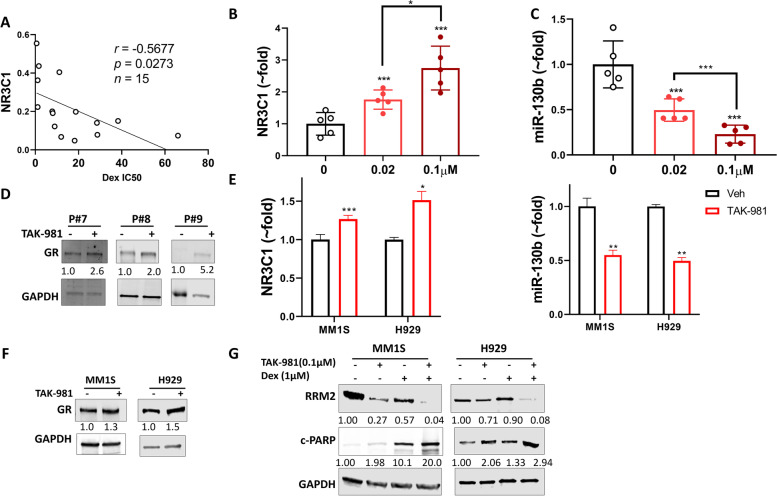
TAK-981 upregulates GR expression in primary multiple myeloma and cell lines through downregulation of miR-130b. **A** GR expression is associated with Dex sensitivity in primary MM cells. GR (NR3C1) mRNA expression was assessed by q-PCR and presented as relative level normalized to GAPDH. IC_50_ values were calculated by GraphPad Prism 8. Pearson correlation analysis showed a significant negative correlation negative correlation between GR RNA (NR3C1) and IC_50_ for Dex in 15 primary MM. **B** NR3C1 mRNA level was decreased and (**C**) miR-130b level was increased in dose-dependent manner in primary MM cells treated with TAK- 981. Primary MM cells from 5 relapsing patients were treated with TAK-981 at indicated concentrations for 48 h. Total RNA extracted and NR3C1 mRNA and miR-130b level was measured by q-PCR. Data presented as mean ± SD, *n* = 5 (**D**) TAK-981 treatment increases GR protein level in primary MM cells. Western blot presents GR protein of 3 primary MM samples treated with or without 0.1 μM TAK-981 for 48 h. Relative protein level was quantified using Image J, normalized to GAPDH, and labeled below each blotting band. **E** NR3C1 mRNA was increased and miR-130b was decreased in MM1S and H929 cell lines upon TAK-981 treatment. Estimated variation is indicated as SD. *P* values were derived using a two-tailed Student *t* test. * *p* < 0.05, ** *p* < 0.01, ***p < 0.001 (**F**) TAK-981 treatment increases GR protein level in MM1S and H929 cell lines. MM1S and H929 cells were treated with or without 20 nM TAK-981 for 48 h. Cell lysates were used for western blot. **G** GR-repressed gene RRM2 and apoptosis marker cleaved PARP (c-PARP) were measured by western blot in MM1S and H929 cell lines treated with TAK-981, Dex or both for 48 h. GAPDH was used as loading controls. Relative protein level was quantified by Image J and labeled below each blot

To evaluate the in vivo effects of TAK-981 on MM tumor growth, we generated two murine xenograft models. NSG mice were i.v. injected with MM1S-Luc cells and tumor burden was determined by mice whole body luminescence imaging. TAK-981 treated mice exhibited a significant lower myeloma burden compared to vehicle group (Fig. 3A-B). In the other model, NSG mice subcutaneously (s.c.) injected with MM1R cells and tumor growth was monitored by tumor volume and weight. Consistent with our in vitro experiment, Dex alone did not affect tumor growth, but TAK-981 treatment greatly suppressed tumor growth and further reduced tumor burden when in combination with Dex (Fig. 3C, Supplemental FigureS3). IHC staining of cleaved PAPR was carried out to determine the apoptosis in tumor tissue. Similarly, Dex treatment alone showed almost no induction of apoptosis, but TAK-981 treatment group exhibited a significant induction of cleaved PARP expression and even higher level of cleaved PARP in combination with Dex (Fig. 2D). The results demonstrated that TAK-981 has potent anti-MM activity in vivo.

### SUMOylation inhibition enhances Dex sensitivity by upregulating GR through downregulating miR-130b

It has been reported that GR expression is a major mechanism of Dex response and is associated with better patient outcome in MM [3, 16, 18]. Consistent with previous studies, we found that GR expression showed negative correlation with Dex IC_50_ values in primary MM cells (*n* = 15) (Fig. 3A). Based on our finding that SUMOylation inhibition enhances Dex sensitivity, we evaluated the effect of SUMOylation inhibition on GR expression. Using primary MM cells obtained from relapsing patients (*n* = 5), we observed a dose-dependent GR (NR3C1) mRNA upregulation upon TAK-981 48 h treatment (Fig. 3B). miR-130b, which has been reported downregulating GR mRNA level by targeting 3′-UTR [19], showed a dose-dependent reduction upon TAK-981 treatment in same primary MM cells (Fig. 3C). GR protein levels were also induced in TAK-981 treated primary MM cells (Fig. 3D). Similar induction of GR level and decrease of miR-130b were observed in MM1S and H929 cell lines (Fig. 3E&F). Transient (siRNA) knockdown of SAE2, UBC9 and stable (shRNA) knockdown of SAE2 both induced NR3C1 mRNA expression and decreased miR-130b levels (Supplemental Fig. S4A-D), indicating the effect of TAK-981 on GR expression is SUMOylation pathway specific. We then evaluated the effects on GR target genes expression. Ribonucleoside-diphosphate reductase subunit M2 (RRM2) has been identified as GR direct target in MM. The expression of RRM2 is repressed by Dex-induced GR activation and the repression of RRM2 is required for Dex-induced apoptosis [20]. TAK-981 decreased RRM2 level and synergistically further reduced the decrease of RRM2 by Dex in both MM1S and H929 cells. There is corresponding induction of apoptosis marker cleaved PARP (Fig. 3G, Supplemental Fig. 4E), indicating the enhancement of TAK-981 to Dex cytotoxicity is related to GR-regulated RRM2 repression. We also observed similar significant changes in the expression of a Dex-activated gene Ras dexamethasone induced 1(RASD1) [21] (Supplemental Fig. 4E), proving the upregulation of GR function upon TAK-981 treatment. To validate our findings, we analyzed public datasets of primary myeloma patient samples. Analysis of cohort (GSE2658) of 559 MM patients (Supplemental Fig. S1B) showed UBA2 level positively correlates with RRM2 and negatively correlates RASD1 expression (Supplemental Fig. S1F). Patients with low SAE2 level (SUMOylation low) showed lower GR-repressed gene RRM2 expression and higher GR-activated gene RASD1 levels. This analysis supports our finding that SUMOylation inhibition upregulates the GR pathway.

Previous studies have shown GR can be SUMO-modified [22–25]. We then evaluated the effect of SUMOylation inhibition on GR activity upon Dex treatment. GR resides in the cytoplasm. Upon ligand (Dex) binding, GR is phosphorylated, then translocates into the nucleus and functions as a transcription factor [26–29]. As we observed TAK-981 48 h treatment increased GR expression level, we carried out a short time TAK-981 treatment to determine GR activity without affecting its expression level. We treated MM1S with TAK-981 for 4 h to fully abolish SUMOylation, then added Dex for 2 h. Dex-induced GR phosphorylation (Ser 211 and Ser 226) and nuclear localization were not affected upon TAK-981 treatment, suggesting SUMOylation regulates GR mainly through mRNA level but not post-translational level (Supplemental Fig. S5).

### SUMOylation inhibition enhances Dex sensitivity in MM through regulating miR-551b and miR-25

Considering inhibition of SUMOylation by TAK-981 and SAE2 knockdown can enhance Dex sensitivity in MM1R cell line among which GR expression is absent, we presume SUMOylation could regulate Dex resistance through other cellular components except GR. Given recently published articles showing that miRNA deregulation may be involved in Dex resistance [4, 30], we conducted miR-seq and RNA-seq in MM1S and MM1R cell lines treated with Dex, TAK-981 or both to identify potential miRs which are involved in Dex resistance and affected by SUMOylation. We cross compared change of miR expression in Dex and TAK-981 treated MM1S and MM1R and identified two candidate miRs: miR-551b and miR-25 (Supplemental Fig. S6).

**Fig. 4:**
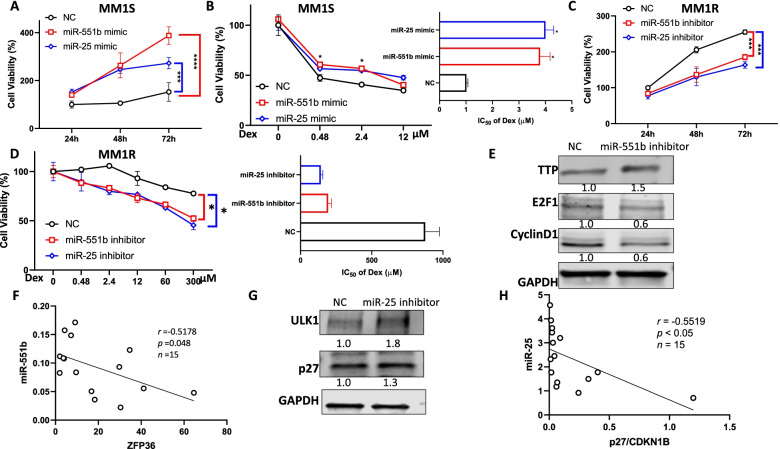
MiR-551b and miR-25 level affect Dex sensitivity in MM. **A** Overexpression of miR-551b and miR-25 increases cell proliferation and (**B**) decreases cell sensitivity to Dex treatment in MM1S cells. (**C**) Knockdown of miR-551b and miR-25 expression inhibits cell proliferation and (**D**) increases sensitivity to Dex in MM1R cells. MM1S cells were transfected with microRNAs: miR-551b mimic, miR-25 mimic or non-targeting control (NC), MM1R cells were transfected with anti-miRs: miR-551b inhibitor, miR-25 inhibitor or non-targeting control (NC). Cell proliferation was measured at 24 h, 48 h and 72 h. Transfected cells were treated Dex at indicated concentration for 48 h. Cell viability was measured and IC_50_ values of Dex were calculated using GraphPad Prism 8 and shown in the right panels. Data were analyzed using unpaired Student *t* tests: Data presented as mean ± SD. ns, not significant; *, *p* < 0.05; **, *p* < 0.01; ***, *p* < 0.001. **E** Knockdown of miR-551b expression increased TTP protein level and decreased E2F1 and CyclinD1 in MM1R cells. **F** miR-551b level negatively correlates with ZFP36 level in in 15 primary MM samples. ZFP36 mRNA expression was assessed by q-PCR and presented as relative level normalized to GAPDH. MiR-551b level was measured by Taqman q-PCR and presented as relative level normalized to RNUB. Pearson correlation analysis was performed. p and r values were labeled. **G** Knockdown of miR-25 expression increased protein levels of ULK1and p27 in MM1R cells. **H** miR-25 level negatively correlates with p27/CDKN1B level in in 15 primary MM samples

To evaluate the role of miR-551b and miR-25 in MM cell growth and Dex resistance, we conducted cell viability assay with overexpression and knockdown of the miRs. Overexpression of miR-551b and miR-25 by transfection of miR mimic promoted MM cell proliferation and reduced Dex sensitivity in MM1S, as IC_50_ increased ~ 4 fold (Fig. 4A-B). Knockdown of miR-551b and miR-25 by transfection of miR inhibitors suppressed cell growth and enhanced Dex sensitivity in MM1R, as IC_50_ decreased from 875 μM to 193 and 142 μM (Fig. 4C-D). Western blot of cleaved-PARP further confirmed the regulation of miR-551b and miR-25 on Dex sensitivity in MM (Supplemental Fig. S7). These data indicated that the expression of miR-551b and miR-25 may contribute to the Dex resistance of MM.

We applied 3 publicly available algorithms Targetscan, miRRDB and miRDIP to predict the potential miR-551b target genes and the results suggests 3 targets: zinc finger protein 36 homolog (ZFP36), praja-1 (PJA1) and zinc finger protein 280C (ZNF280C) (Supplementary Fig. S8). Because PJA1 and ZNF280C are not abundantly expressed in MM cells, ZFP36 became the only potential target of miR-551b. ZFP36-encoded protein, tristetraprolin (TTP), an RNA binding protein, plays an important role in cancer cell proliferation through destabilizing Cyclin D1 and E2F1 mRNA [31–34]. Knockdown of miR-551b level by transfection of miR-551b inhibitor caused the increase of TTP level and decrease of Cyclin D1 and E2F1 (Fig. 4E). The expression of ZFP36 is negatively correlated with miR-551b in primary MM samples (Fig. 4F), further indicating miR-551b regulating ZFP36 level.

MiR-25 has been characterized with onco-miR function and has multiple validated targets including Unc-51 Like Autophagy Activating Kinase 1 (ULK1) and Cyclin-dependent kinase inhibitor 1B CDKN1B(p27, 35–39]. Knockdown of miR-25 significantly increased ULK1 levels. Knockdown of miR-25 caused an increased level of p27, which is consistent with the observation that p27 mRNA level is negatively correlated with miR-25 in primary MM samples (Fig. 4G-H).

**Fig. 5:**
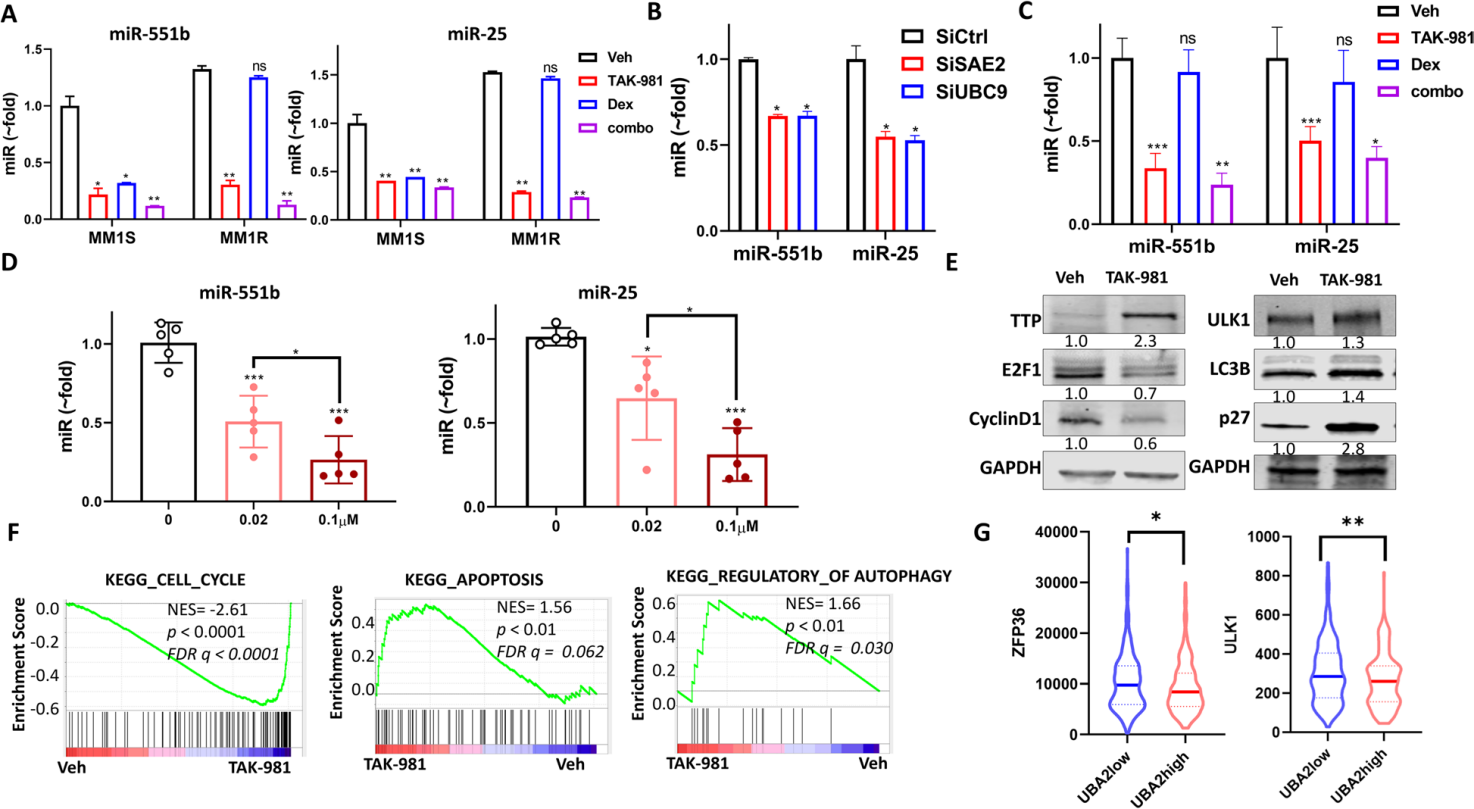
SUMOylation inhibition decreases miR-551b and miR-25 level. **A** miR-551b and miR-25 levels were decreased by TAK-981 treatment in MM1S and MM1R cells. MM1S and MM1R cells were treated with Vehicle (Veh), 0.1 μM TAK-981 (TAK), 1 μM Dex (Dex), or 0.1 μM TAK-981 with 1 μM Dex (combo) for 48 h. miR level was measured by Taqman q-PCR. **B** Knockdown of SAE2 or UBC9 decreases the expression of miR-551b and miR-25. MM1R cells were transfected with siRNA targeting SAE2 (SiSAE2) or UBC9 (SiUBC9), or non-targeting control (SiCtrl) for 72 h, miR levels were measured by qPCR. **C** TAK-981 treatment decreases miR-551b and miR-25 levels in vivo. Tumor tissue from MM1R xenograft mice (in Fig. 2C) was used for RNA extraction and miR measurement. **D** TAK-981 treatment decreased miR-551b and miR-25 level in primary MM cells in dose-dependent manner in primary MM cells treated with TAK- 981. Primary MM cells from 5 relapsing patients were treated with TAK-981 at indicated concentrations for 48 h. miR-551b and miR-25 level was measured by q-PCR. Data presented as mean ± SD, *n* = 5. **E** TAK-981 treatment affects miR-551b and miR-25 downstream genes protein level. Western blot presents protein level in MM1R cells treated with vehicle (Veh) and 0.1 μM TAK-981 (TAK-981) for 48 h. Relative protein level was quantified by Image J and labeled below each blot. **F** GSEA shows cell cycle regulators are suppressed but apoptosis and autophagy related pathways are upregulated upon TAK-981 treatment. GSEA false discovery rate (FDR)-q values, *p*-values and normalized enrichment scores (NES) are labeled. **G** UBA2 level correlates with ZFP36 and ULK1 expression in patient specimens. Analysis of cohort (GSE2658) of 559 MM patients (Supplemental Fig. S1B). Patients with high SAE2 (UBA2; UBA2high group) showed lower ZFP36 levels (left) and ULK1 level (right) than patients with low SAE2 (UBA2; UBA2low group). Data were analyzed using unpaired Student t tests: Data presented as mean ± SD. ns, not significant; *, *p* < 0.05; **, *p* < 0.01; ***, *p* < 0.001

MM1R cells exhibit higher level of miR-551b and miR-25 than MM1S (Fig. 5A, Supplemental Fig. S6). Dex treatment decreased these two miRs levels in MM1S but showed no effect in MM1R, but TAK-981 treatment decreased them in both MM1S and MM1R cell lines and showed further reduction in combination with Dex (Fig. 5A, Supplemental Fig. S9A). Knockdown of SAE2 or UBC9 by siRNA transient transfection decreased miR-551b and miR-25 levels in MM1R (Fig. 5B). Stable knockdown (shSAE2) of SAE2 exhibited reduced level of miR-551b and miR-25 in RPMI-8226 cell line (Supplemental Fig. S9B). To confirm that miR-551b and miR-25 expression is regulated by SUMOylation in vivo, we examined tumor tissue from MM1R xenograft and primary MM samples. Consistent with cellular experiment results, miR-551b and miR-25 levels were reduced in TAK-981 treatment group and further decreased in combination with Dex treatment in MM1R xenograft tumor tissue (Fig. 5C). We treated 5 primary MM samples with different concentration of TAK-981, miR-551b and miR-25 levels significantly decreased in a dose-dependent manner, indicating that the regulation of miR-551b and miR-25 by SUMOylation is not restricted to cell lines (Fig. 5D).

We then evaluated the impact of SUMOylation inhibition on target gene levels of these miRs. TAK-981 treatment caused induction of miR-551b target gene TTP expression and resulted in Cyclin D1 and E2F1 decrease in MM1R cells (Fig. 5E). Because E2F1 is a transcription factor, GSEA analysis was performed and results indicated that E2F1 target gene sets were suppressed upon TAK-981 treatment (Supplemental Fig. S9C). TAK-981 treatment induced expression of miR-25 target genes ULK1 and p27(Fig. 5E). mRNA levels of ZFP36(TTP), ULK1 and p27 showed reversed changes compared to miR-551b and miR-25 in Dex, TAK-981 and combo treated MM1S and MM1R cells (Supplemental Fig. S9D). The expression levels were increased upon TAK-981 treatment and further enhanced in combination with Dex. Furthermore, TAK-981 caused induction of ZFP36 mRNA level was suppressed by exogenous expression of miR-551b. The increased ULK1 and p27(CDKN1B) mRNA levels upon TAK-981 treatment were also suppressed by overexpression of miR-25, indicating that SUMOylation inhibition increased ZFP36 through miR-551b and regulated ULK1 and p27 through miR-25 (Supplemental Fig. S9E). ULK1 is a major enzyme in autophagy pathway, Cyclin D1 and p27 play important roles in cell cycle regulation and apoptosis. GSEA analysis of RNA-seq indicated TAK-981 treatment affected cell cycle, apoptosis and autophagy gene sets (Fig. 5F, Supplemental Table S2), suggesting the effects may be achieved through these genes targeting microRNAs- miR-551b and miR-25 level. We analyzed a MM patients data set GSE2658 (*n* = 559), among which SAE2(UBA2) level is associated with poor outcome (Supplemental Fig. S1B). Patients with low SAE2 (UBA2; UBA2low group) showed higher ZFP36 (miR-551b target gene) and ULK1 (miR-25 target gene) levels than patients with high SAE2 (UBA2; UBA2high group) (Fig. 5G). The analysis further supports our finding that SUMOylation regulates miR-551b, miR-25 and their target genes expression.

### SUMOylation inhibition decreases the expression miR-551b, miR-25 and miR-130b via c-Myc

Next, we investigated whether SUMOylation inhibition caused decrease of miR-551b, miR-25 and miR-130b levels through mediating the transcription of pri-miRNAs. Upon TAK-981 treatment, pri-miR-551b, pri-miR-25 and pri-miR-130b expression levels significantly decreased in both MM1S and MM1R cells (Supplemental Fig. S10A). Knockdown of SAE2 in RPMI8226 cells caused similar reduction of these pri-miRs (Supplemental Fig. S10B). Therefore, these results indicate SUMOylation regulates miR-551b, miR-25 and miR-130b gene expression at transcriptional level.

**Fig. 6:**
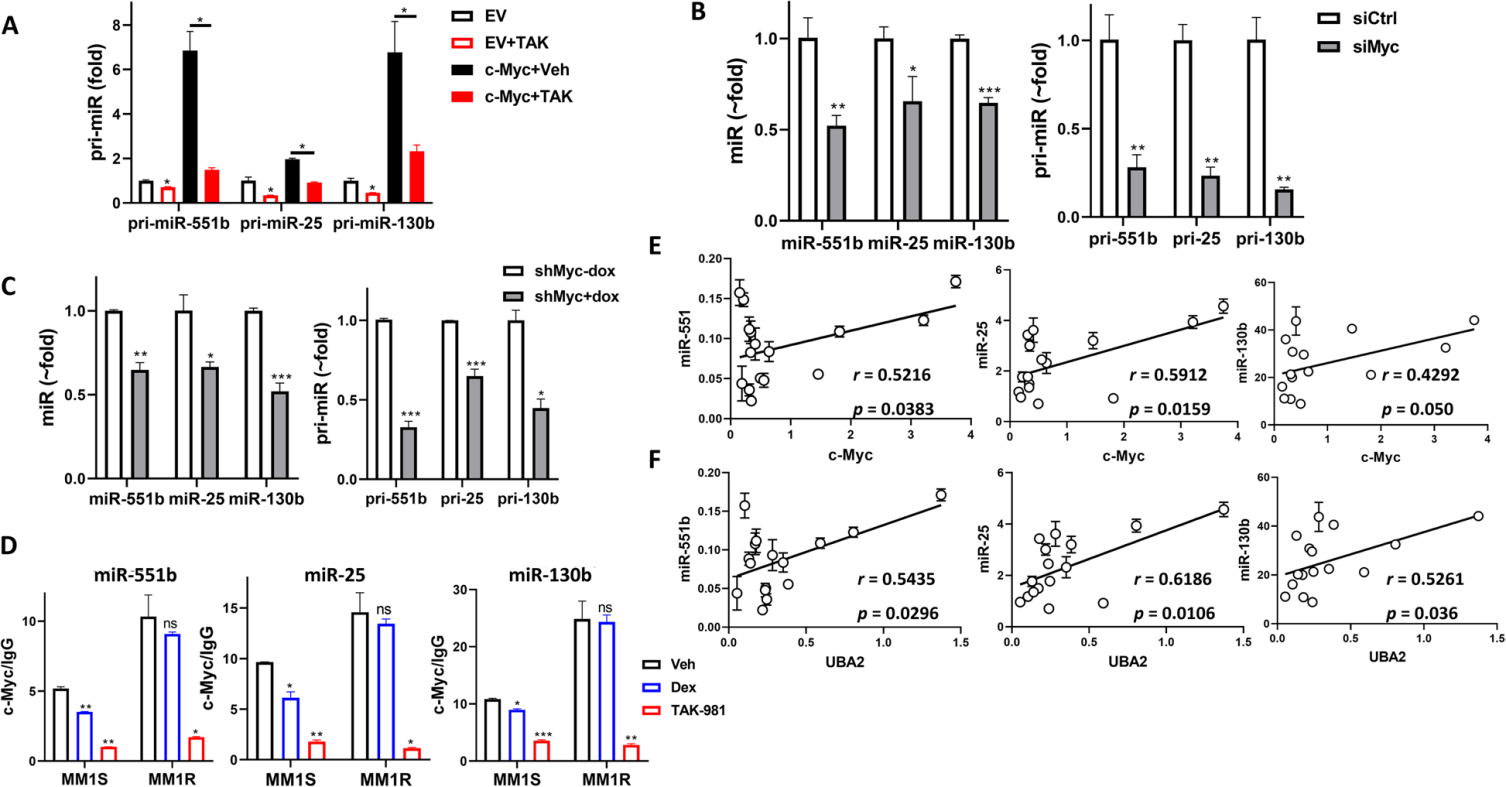
c-Myc is a major transcription factor of miR-551b, miR-25 and miR-130b. **A** Overexpression of c-Myc induced pri-miR-551b, pri-miR-25 and pri-miR-130b and the induction can be inhibited by TAK-981 treatment. MM1S cells were transfected with empty vector (EV) or c-Myc expression plasmid (c-Myc) then treated with 0.1 μM TAK-981 (TAK) or vehicle (Veh) for 24 h. Pri-miR level was measured by q-PCR. **B** Knockdown of c-Myc decreased miR-551b, miR-25 and miR-130b miR and pri-miR level. MM1S cells were transfected with siRNA targeting c-Myc (siMyc) or non-targeting control (SiCtrl) for 72 h, miR and pri-miR level were determined by qPCR. **C** RPMI8226 stable cell line was generated with inducible shRNA targeting c-Myc. Dox (5 μg/mL) was added to induce c-Myc knockdown (shMyc+Dox) for 48 h. miRs and pri-miRs levels were measured by qPCR. **D** ChIP assay identified c-myc binding at the promoter regions of miR-551b, miR-25, and miR-130b. TAK-981 treatment decreases the c-Myc binding occupancy in both MM1S and MM1R cells but Dex only decreases in MM1S cells. ChIP was performed using an anti-c-Myc antibody in MM1S and MM1R cells treated with Vehicle (Veh), 0.1 μM TAK-981 (TAK-981) or 1 μM Dex (Dex). The occupancy was normalized to DNA input and calculated relative to IgG control. Data were analyzed using ANOVA. Data presented as mean ± SD. ns, not significant; *, *p* < 0.05; **, *p* < 0.01; ***, *p* < 0.001. **E** c-Myc and (**F**) SAE2 (UBA2) level correlates with miR-551b, miR-25 and miR-130b level in 15 primary MM samples. Pearson correlation analysis was performed. p and r values were labeled

To identify the transcription factors involved in regulating miR-551b, miR-25 and miR-130b expression in a SUMOylation-dependent manner, we focused on the transcription factors E2F1, c-Fos, and c-Myc, which were shown to bind to the promoter regions in genome-wide ChIP-seq studies using UCSC Genome Browser (genome.ucsc.edu) [35, 36]. We transfected plasmids expressing E2F1, c-Fos, or c-Myc into 293 T cells, then measured pri-miRs level by qPCR. All three pri-miR levels were significantly increased by overexpression of c-Myc, but were not affected by E2F1 or c-Fos overexpression (Supplemental Fig. S10C). The promoter of miR-551b was subcloned to luciferase reporter plasmid, and miR-551b promoter activity was significantly increased by c-Myc overexpression in a dose-dependent manner (Fig. S10D). Because miR-25 promoter is co-localized with miR-93 and miR-106b and miR-130b promoter is in the same region as miR-301b, the reporter plasmid and assay of miR-25 and miR-130b promoter were not conducted. In MM1S cells, c-Myc induced increase of pri-miR levels of miR-551b, miR-25 and miR-130b can be inhibited by addition of TAK-981 treatment (Fig. 6A). In order to further determine the role of c-Myc on miR-551b, miR-25 and miR-130b expression, knockdown of c-Myc expression was carried out. siRNA transfection of c-Myc (siMyc) in MM1S cells caused significant reduction of pri-miRs and mature miR levels (Fig. 6B). We generated stable cell line that can be induced to express shRNA targeting c-Myc in RPMI8226 cells. The pri-miRs and mature miRs decreased after Dox-induced c-Myc knockdown (Fig. 6C and Supplemental Fig. S10E). All these results demonstrated c-Myc as a major transcriptional activator of miR-551b, miR-25 and miR-130b gene expression. Next we evaluated the binding of endogenous c-Myc to miR-551b, miR-25 and miR-130b promoters using ChIP in MM1S and MM1R cells treated with TAK-981 or Dex. TAK-981 treatment significantly decreased c-Myc binding occupancy at the promoter regions of these miRs in both MM1S and MM1R cells (Fig. 6D), proving SUMOylation regulates these miRs transcription via c-Myc binding to the promoter region. Dex showed minor decrease of c-Myc binding in MM1S cells and no effects in MM1R cells, indicating these miRs are involved in Dex sensitivity in MM.

The regulation of c-Myc and SAE2 of miR-551b, miR-25 and miR-130b in MM cell was further confirmed using qPCR by observing a significant positive correlation between c-Myc and SAE2 level and the expression of these miRs in primary MM cells from 15 relapsed patients (Fig. 6E-F). All these demonstrate that SUMOylation regulates miR-551b, miR-25 and miR-130b expression via mediating c-Myc.

### SUMOylation inhibition decreases c-Myc level

**Fig. 7:**
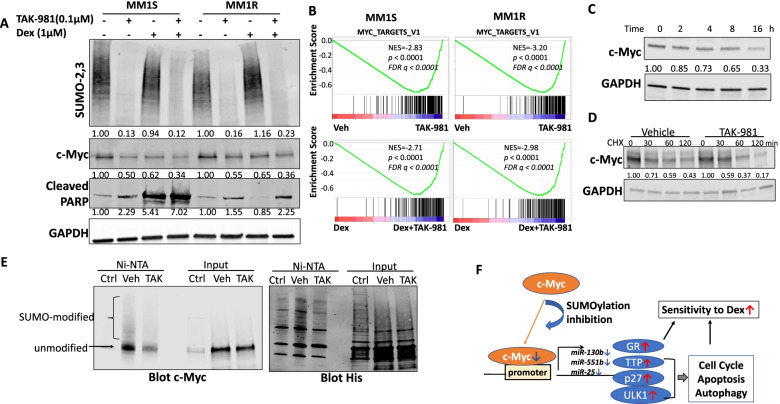
SUMOylation inhibition decreased c-Myc protein level through regulating protein stability. **A** TAK-981 treatment decreases c-Myc levels in both MM1S and MM1R cell lines and Dex has no effect on c-Myc level in MM1R cell line. MM1S and MM1R cells were treated with TAK-981 or Dex or both for 48 h, c-Myc and cleaved PAPR (c-PARP) levels were determined by western blot. SUMO-2,3 was blotted to determine global SUMOylation and GAPDH was used as loading control. **B** GSEA analysis of RNA-seq data shows TAK-981 treatment inhibits c-Myc target gene sets in MM1S and MM1R cells. **C** TAK-981 treatment decreases c-Myc protein level in time-dependent manner. MM1R cells were treated with TAK-981 at 0.1 μM and cells were harvested at indicated time points. C-Myc protein level was determined by western blot. **D** TAK-981 treatment accelerates c-Myc protein degradation. MM1R cell pre-treated with vehicle or TAK-981 for 2 h were exposed to cycloheximide (CHX) to block protein synthesis, cells were harvested at indicated time points and analyzed for c-Myc level by western blot. c-Myc level was quantified by ImageJ, normalized to GAPDH and plotted. **E** SUMO modification of c-Myc was reduced upon TAK-981 treatment. 293 T cells were transfected with His-tagged SUMO-1, His-tagged SUMO-2 and untagged c-Myc expression plasmids. Cells were treated with 0.1 μM TAK-981(TAK) or Vehicle (Veh) for 8 h. Cell lysates were incubated with Ni-NTA beads to pull down all His-tagged SUMO-1 and SUMO-2, followed by Western blot analysis with c-Myc antibody (left) or an anti-His-tag antibody (right). Cells transfected with His-tagged SUMO-1 and SUMO-2 but without c-Myc expressing plasmid were used as control (Ctrl). SUMO-modified and unmodified c-Myc were pointed. **F** Schematic showing the mechanism of SUMOylation inhibition enhance MM sensitivity to Dex. SUMOylation inhibition decreases c-Myc level, causes reduction of miR-130b, miR-551b and miR-25. The decrease of miR-130b results in induction of GR expression. miR-551b and miR-25 reduction causes increase of TTP, ULK1, p27, resulting in dysregulation of cell cycle, apoptosis and autophagy. All these contribute to enhanced sensitivity to Dex

We then evaluated the impact of SUMOylation inhibition on c-Myc level. Dex decreased c-Myc protein in Dex-sensitive MM1S cells, but showed no effect in MM1R cells. However, TAK-981 treatment showed reduction of c-Myc protein in both MM1S and MM1R cell lines and caused further decrease when combined with Dex (Fig. 7A). Consistent with this, c-Myc level decreased when SAE2 was knockdown in RPMI-8226 cells (Supplemental Fig. S11A). The reduction of TAK-981 on c-Myc level was also observed in another MM cell line H929 (Supplemental Fig. S11B). IHC staining in tumor tissues from MM1R xenograft mice indicated TAK-981 decreased c-Myc level and further reduced when combined with Dex although Dex alone showed no effect on c-Myc in vivo (Supplemental Fig. S11C). GSEA indicated TAK-981 treatment suppressed Myc target gene sets in both MM1S and MM1R cells, TAK-981 and Dex combination treatment showed further suppression of Myc targets compared to Dex treatment alone (Fig. 7B, Supplemental Fig. 11D). We observed a time-dependent decrease of c-Myc protein level upon TAK-981 treatment in MM1R cells (Fig. 7C). Because c-Myc has been identified as SUMO-modified protein [37, 38], we measured protein stability using a cycloheximide (CHX) assa. c-Myc decreased faster in myeloma cells treated with TAK-981 compared to vehicle (Fig. 7D, Supplemental Fig. S11E), indicating SUMOylation inhibition downregulated c-Myc by enhancing their degradation. We performed IP assays to confirm the SUMO modification of c-Myc was reduced by SUMOylation inhibition or SAE2 knockdown. 293 T cells were transfected with His-tagged SUMO-1, His-tagged SUMO-2 and untagged c-Myc expression plasmids. Cells were treated with 0.1 μM TAK-981(TAK) or Vehicle (Veh) for 8 h then Cell lysates were harvested and incubated with Ni-NTA beads to pull down all His-tagged SUMOs then blot c-Myc level (Fig. 7E). The western blot indicated there is much less SUMO-modified c-Myc upon TAK-981 treatment. We conducted IP assay to detect SUMO-modification of endogenous c-Myc in RPMI8226 stable cell line with inducible SAE2 shRNA. The results indicated there was less SUMO modification of c-Myc upon SAE2 knockdown (Supplemental Fig. S11F). These results, along with literature, proved the regulation of c-Myc by direct SUMO-modification.

We also observed c-Myc mRNA level decreased upon TAK-981 treatment, which is consistent with our previous finding that SUMOylation regulates c-Myc mRNA level through regulation its targeting microRNA miR-34b/c [39].

Except MM, Glucocorticoids (GCs) are a mainstay of treatment of lymphoid malignancies including Acute Lymphoblastic Leukemia (ALL). We treated Jurkat, a T-ALL cell line, with TAK-981, Dex or both, then measured cell viability. Consistent with previous studies [40, 41], Jurkat cells are resistant to Dex treatment, TAK-981 treatment alone showed potent cytotoxicity and further enhanced cell death when combined with Dex (Supplemental Fig. S12A). Apoptosis assay and cleaved-PARP level further confirmed that TAK-981 enhanced Dex sensitivity in Jurkat cells (Supplemental Fig. S12B-C). Similar as we observed in MM, TAK-981 treatment increased GR and decreased c-Myc level, along with downregulation of miR-130b, miR-551b and miR-25 in Jurkat cells (Supplemental Fig. S12C-E). These findings suggest the mechanism of SUMOylation inhibition enhances Dex sensitivity might not be restricted in MM but also applicable to other types of hematologic cancer.

## Discussion

Resistance to Dex therapy is associated with poor prognosis in MM. Our study demonstrated SUMOylation plays an important role in Dex resistance. The expression level of SUMO E1 (SAE2) was inversely correlated with Dex sensitivity of primary MM samples. SUMOylation inhibition by decreasing SUMO E1 level via shRNA knockdown or inhibiting SUMO E1 activity by TAK-981 greatly enhanced MM sensitivity to Dex treatment. SUMOylation inhibition reduced myeloma cell viability and induced apoptosis. TAK-981 showed potent cytotoxicity against primary relapsing MM samples and in vivo. Moreover, combination TAK-981 with Dex showed potent synergistic anti-MM effects ex vivo and in vivo (Fig. 1-2), supporting translation of this SUMO E1 inhibitor into myeloma trials.

Our study uncovered a mechanism in which SUMOylation inhibition reduces c-Myc level, leads to decreased expression of miR-130b, resulting in upregulation of GR level. This upregulation greatly enhances Dex sensitivity via affecting expression of downstream genes RRM2 and RASD1, leading to synergistical cytotoxicity (Figs. 3 and 7). Noticeably, although SUMOylation inhibition induced GR level only ~ 1.5 fold, it caused dramatic changes of RRM2 and RASD1 levels, indicating the effect on the whole GR pathway. Our group has reported miR-130b can downregulate GR by targeting 3′-UTR [19], the current study further revealed a regulatory machinery of this micro RNA.

Our study identified miR-551b and miR-25 as important miRs mediating Dex resistance in MM. miR-551b and miR-25 levels are higher in Dex-resistant cell line MM1R than Dex-sensitive cell line MM1S. Overexpression of miR-551b and miR-25 caused resistance to Dex treatment in MM1S, however, knockdown of miR-551b and miR-25 significantly enhanced Dex sensitivity in MM1R (Fig. 4). We identified ZFP36 as the target gene of miR-551b. ZFP36 encodes an RNA-binding protein, TTP, which downregulates two key factors in myeloma growth and proliferation - CyclinD1 and E2F1. Knockdown of miR-551b caused increased TTP, led to less Cyclin D1 and E2F1 level, resulting in decreased myeloma proliferation and enhanced Dex sensitivity. MiR-25 has been characterized with onco-miR function with multiple validated target genes [42–46]. We demonstrated miR-25 mediates Dex resistance in myeloma via targeting ULK1 and p27. ULK1 is an essential enzyme in autophagy pathway. It has been reported that Dex induced pro-death autophagy in MM cells, and the inhibition of autophagy significantly decreased Dex-induced cell death [4]. P27 has a broad inhibitory activity against different CDKs and reduce cell proliferation by cell cycle arrest, acting as a potential tumor suppressor gene. Knockdown of miR-25 increased ULK1 and p27 level, upregulating autophagy and decreasing cell proliferation, contributing to Dex sensitivity in MM. There are recent publications showing that miRNA deregulation may be involved in Dex resistance [4, 30]. Our study identified two novel miRNAs promoting Dex resistance independent of GR pathway, providing new insights into understanding of Dex resistance mechanism.

SUMOylation inhibition or SAE2 knockdown decreased miR-551b and miR-25 levels in MM cell lines and primary relapsing patient samples. TAK-981 treatment phenocopies the effects on target genes (TTP/CylinD1/E2F1, ULK1/p27) by knockdown of miR-551b and miR-25, suggesting SUMOylation inhibition enhances Dex sensitivity via decreasing these two miRs. The correlation of SAE2 with ZFP36 and ULK1 in patient cohorts further supports the regulation of SUMOylation on miR-551b and miR-25 (Fig. 5). A previous study reported that MiR-221/222 promotes Dex resistance in MM [4]. We measured miR-221/222 level upon SUMOylation inhibition as well. We observed TAK-981 decreased miR-221/222 level but the reduction is much less potent than the effect on miR-551b or miR-25. So we focus on miR-551b and miR-25.

Our study, combined with previous studies, reveals that SUMOylation regulates miR-551b, miR-25 and miR-130b through modulation of c-Myc levels. We demonstrated c-Myc is a major transcriptional activator of these miRs by qPCR upon overexpression or knockdown of c-Myc and ChIP assay upon TAK-981 or Dex treatment. The regulation of c-Myc on these miRs was further validated by the positive correlation of c-Myc with expression of miR-551b, miR-25 and miR-130b in primary MM samples (Fig. 6). Our findings revealed a SUMO-dependent regulation of c-Myc, the key transcriptional factor of several miRs which mediates Dex resistance in MM.

We found TAK-981 treatment decreased c-Myc level, led to decreased miR-551b and miR-25 level, resulting in apoptosis and autophagy, contributing to Dex sensitivity in MM (Fig. 7). We observed TAK-981 treatment enhances c-Myc protein degradation in MM. As we also observed the decrease of c-Myc mRNA level, which is consistent with our previous study that SUMOylation regulates c-Myc mRNA level through regulation its targeting microRNA miR-34b/c [39], we believe SUMOylation regulates c-Myc not only through post-transcription via microRNA targeting, but also through post-translation via regulating protein stability.

Dex is a mainstay of treatment of lymphoid malignancies and resistance to GCs is the single most reliable prognostic indicator for relapse among children with ALL [47]. Our investigation demonstrated TAK-981 showed potent cytotoxicity and a synergistic effect with Dex against Jurkat cells. Similar as in MM, SUMOylation inhibition caused upregulation of GR expression and downregulation of miR-551b and miR-25 level (Supplemental Fig. S12). These data suggest the effects and mechanism of SUMOylation inhibition enhancing Dex sensitivity in MM might be applicable to other hematologic malignancies.

## Conclusion

Together, our study revealed that SUMOylation inhibition enhances Dex sensitivity in MM by increasing GR, a previously identified key factor regulating Dex resistance and decreasing miR-551b and miR-25, two microRNAs which we confirmed for the first time as Dex resistance mediators in our investigation. SUMO E1 inhibitor TAK-981 greatly enhanced Dex sensitivity in MM cell lines, primary samples and mouse xenograft models. Combination TAK-981 with Dex showed potent synergistic anti-MM effects ex vivo and in vivo. Our findings strongly support translation of TAK-981 into clinical trials for MM patients, and possibly other hematologic malignancies.

## Supplementary Information

**Additional file 1.**

## Data Availability

RNA-seq and miRNA-seq data have been deposited to the GEO database (GSE 163682). All other data are available in the main text or the supplementary materials.

## Abbreviations

Dex: Dexamethasone
MM: Multiple myeloma
SUMO: Small ubiquitin-like modifier
UBA2: Ubiquitin Like Modifier Activating Enzyme 2
SAE2: SUMO activating enzyme 2
ChIP: Chromatin immunoprecipitation
GC: Glucocorticoid
GR: Glucocorticoid receptor
IHC: Immunohistochemistry
RASD1: Ras dexamethasone induced 1
RRM2: Ribonucleoside-diphosphate reductase subunit M2
TTP: Tristetraprolin
ZFP36: Zinc finger protein 36 homolog
PJA1: Praja-1 (PJA1)
ZNF280C: Zinc finger protein 280C
ULK1: Unc-51 Like Autophagy Activating Kinase 1
CDKN1B/p27: Cyclin-dependent kinase inhibitor 1B
GSEA: Gene set enrichment analysis
ALL: Acute Lymphoblastic Leukemia
