# Supplementary material for: SUMOylation inhibition enhances dexamethasone sensitivity in multiple myeloma
Source: J Exp Clin Cancer Res. 2022 Jan 4;41:8. doi: 10.1186/s13046-021-02226-9 (PMC8725350; doi:10.1186/s13046-021-02226-9)
Supplement: Supplementary file 1 — Additional file 1. [file 13046_2021_2226_MOESM1_ESM.pdf]

Supplementary Methods

Primers for qRT-PCR, promoter clone and ChIP analysis

For qRT-PCR using Sybr green: NR3C1 forward, 5'- TGCCGCTATCGAAAATGTCTT -3'; NR3C1 reverse, 5'- GGGTAGGGGTGAGTTGTGGT -3', UBA2 forward, 5'- CCCGAAAGCTAATATCGTTGCC -3', UBA2 reverse, 5'- ACTCGGTCACACCCTTTTTGA -3', UBC9 forward, 5'- ACCATTATTTCACCCGAATGTGT -3', UBC9 reverse, 5'- CTCGGACCCTTTTCTCGTACT -3', RASD1 forward, 5'- AGCTGAGTATCCCGGCCAA -3', RASD1 reverse, 5'- CGATGGTAGGCGTGTAGGC-3', RRM2 forward, 5'- GTGGAGCGATTTAGCCAAGAA-3', RRM2 reverse, 5'- CACAAGGCATCGTTTCAATGG-3', ZFP36 forward, 5'- GACTGAGCTATGTCGGACCTT-3', ZFP36 reverse, 5'- GAGTTCCGTCTTGTATTTGGGG-3', ULK1 forward, 5'- AGCACGATTTGGAGGTCGC-3', ULK1 reverse, 5'- GCCACGATGTTTTCATGTTTCA-3', CDKN1B forward, 5'- AACGTGCGAGTGTCTAACGG-3', CDKN1B reverse, 5'- CCCTCTAGGGGTTTGTGATTCT-3', cMyc forward, 5'- GTCAAGAGGCGAACACACAAC-3', cMyc reverse, 5'- TTGGACGGACAGGATGTATGC-3', GAPDH forward, 5'- AGGTCGGAGTCAACGGATTTG-3'; and GAPDH reverse,5'- GTGATGGCATGGACTGTGGT-3'.

For qRT-PCR using Taqman probe: miR and pri-miR probes are purchased from Invitrogen. miRs—miR-551b (Assay ID 002346), miR-25 (Assay ID 002442), miR-130b (Assay ID 000456) and RNU6B (Assay ID 001093). Pri-miRs – pri-miR-551b (Hs03304341_pri), pri-miR-25 (Hs04227110_pri), pri-miR-130b (Hs03303416_pri) and GAPDH (Hs02786624_g1).

For cloning the promoter of miR-551b to pGL2.1-Luciferase reporter vector, forward 5'- ATTGGTACCCCCATGAGTCCTCGTGCATT-3', reverse 5'- ACTAAGCTTAGTGACTTGCGTCAGCTCAA-3'.

For ChIP assay, PCR primers specific for the c-Myc binding sites on the miR-551b promoter: forward, 5'- ATGGCAAATGCGTGACTACC-3', reverse, 5'- TCTAGAGCTGTGAGCGCAAAA-3', miR-25 promoter, forward, 5'-

CGGAAGGACACTGTTTACACG-3’, reverse, 5’- CTTTTGGGCATTCCCAGGTT-3’, miR-130b promoter, forward, 5’- CTGAGAAAAGGCGGGCAAAG-3’, reverse, 5’- CCCCGACTCCTTAGCATAGC-3’.

shRNA sequences

All shRNAs were purchased from GE Dharmacon.

ShSAE2#1 (V2THS_254939): ATAGCAGGAATAATGTTCC

ShSAE2#5(V2THS_68114): TCAATAAGAGGAACATCAG

Myc shRNA(V2THS_152051): TTGTGTTTCAACTGTTCTC

Table S1. TAK-981 synergizes with Dex in decreasing cell viability in primary multiple myeloma cells from patient specimen. Combination Index (CI) determined by CompuSym.

Specimen ID	TAK-981(μM)	Dex (μM)	CI
P#1	0.016	0.08	0.319
	0.08	0.4	0.032
	0.4	2.0	0.042
	10.0	50.0	0.148
	2.0	10.0	0.050
	50.0	250.0	0.037
P#2	0.016	0.08	0.038
	0.08	0.4	0.071
	0.4	2.0	0.050
	10.0	50.0	0.147
	2.0	10.0	0.179
	50.0	250.0	0.080
P#3	0.0064	0.032	0.431
	0.08	0.4	0.020
	0.4	2.0	0.019
	2.0	10.0	0.048
	10.0	50.0	0.079
	50.0	250.0	0.039
P#4	0.032	0.16	0.122
	0.16	0.8	0.194
	0.8	4.0	0.921
	100.0	500.0	0.003
P#5	0.032	0.16	0.000
	0.16	0.8	0.000
	0.8	4.0	0.000
	4.0	20.0	0.000
	20.0	100.0	0.001
	100.0	500.0	0.000
P#6	0.0064	0.032	0.013
	0.032	0.16	0.052
	0.16	0.8	0.159
	0.8	4.0	0.454
	4.0	20.0	0.969
	100.0	500.0	0.040

Table S2. Top ranked hallmark gene sets identified from gene expression data in MM1S cells exposed to TAK-981.

Downregulated

Gene Sets	NES	NOM p-value	FDR q-value
HALLMARK_E2F_TARGETS	-3.28	0.0000	0.0000
HALLMARK_G2M_CHECKPOINT	-2.99	0.0000	0.0000
HALLMARK_MYC_TARGETS_V1	-2.83	0.0000	0.0000
HALLMARK_MTORC1_SIGNALING	-2.58	0.0000	0.0000
HALLMARK_MYC_TARGETS_V2	-2.36	0.0000	0.0000
HALLMARK_MITOTIC_SPINDLE	-2.20	0.0000	0.0000
HALLMARK_GLYCOLYSIS	-2.11	0.0000	0.0000
HALLMARK_SPERMATOGENESIS	-2.09	0.0000	0.0000
HALLMARK_ESTROGEN_RESPONSE_LATE	-2.08	0.0000	0.0000
HALLMARK_UNFOLDED_PROTEIN_RESPONSE	-1.95	0.0000	0.0001

Upregulated

Gene Sets	NES	NOM p-value	FDR q-value
HALLMARK_INTERFERON_ALPHA_RESPONSE	2.88	0.0000	0.0000
HALLMARK_INTERFERON_GAMMA_RESPONSE	2.71	0.0000	0.0000
HALLMARK_TNFA_SIGNALING_VIA_NFKB	2.02	0.0000	0.0000
HALLMARK_INFLAMMATORY_RESPONSE	1.78	0.0000	0.0029
HALLMARK_KRAS_SIGNALING_DN	1.75	0.0023	0.0028
HALLMARK_COMPLEMENT	1.65	0.0000	0.0065
HALLMARK_IL6_JAK_STAT3_SIGNALING	1.61	0.0116	0.0081
HALLMARK_ALLOGRAFT_REJECTION	1.46	0.0080	0.0342
HALLMARK_P53_PATHWAY	1.40	0.0051	0.0460

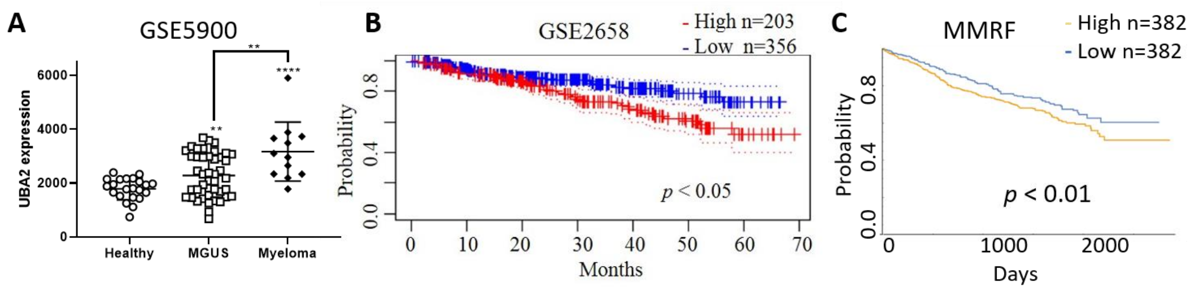

Supplementary Figure S1. The expression level of SUMO E1 is upregulated in Multiple Myeloma and associated with poor prognosis. (A) Expression level of SUMO E1, UBA2 in healthy plasma cells (Healthy, n= 22) premalignant myeloma stage (MGUS, n=44) and in the asymptomatic myeloma phase (Myeloma, n=12) in datasets GSE5900. (B) UBA2 level is associated with poor prognosis in MM cohort GSE2658 (n=559). *P* values were derived using a two-tailed Student *t* test. **, $P < 0.01$; ****, $P < 0.0001$ (B) and CoMMpass (n=764) (C). (B)Kaplan–Meier plots were generated using online server Prognoscan for GSE2658. Solid lines represent two patient groups: high SAE2 level group (red) and low SAE2 level group (blue). Dotted lines indicate the 95% confidence intervals for each group. (C) Kaplan–Meier plots from CoMMpass MMRF (clinical trial NCT01454297). Patients were divided to two groups by median value of UBA2 expression: high UBA2 level group (yellow line) and low SAE2 level group (blue line). P-values are labeled as indicated.

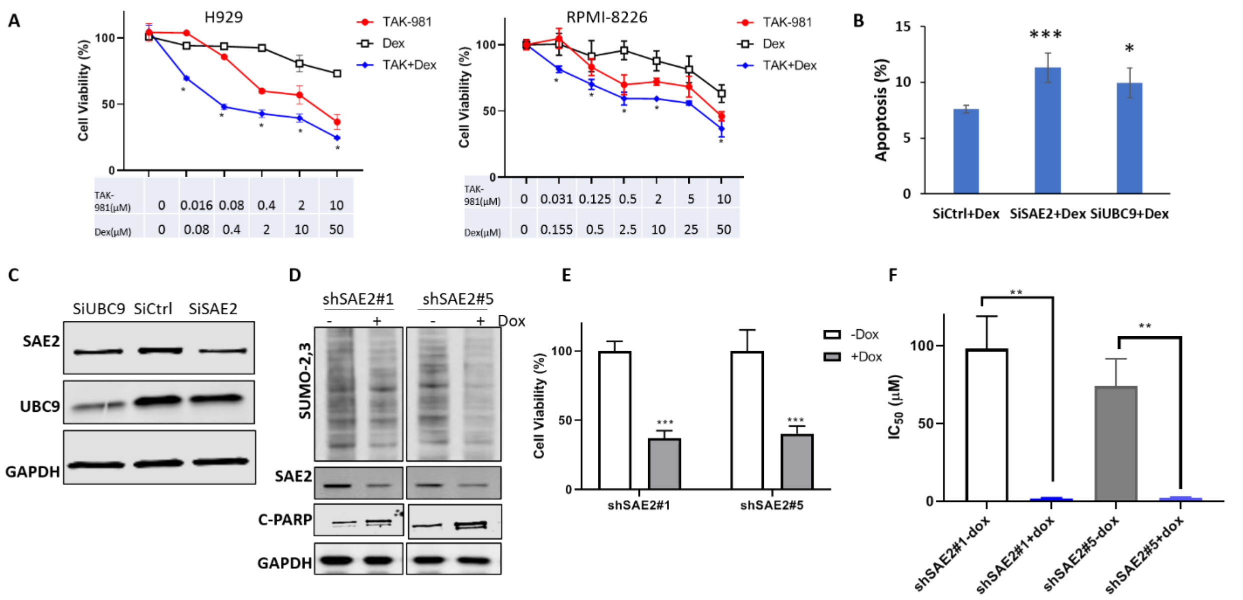

Supplementary Figure S2. SUMOylation inhibition enhances Dex sensitivity in MM. (A) TAK-981 synergizes with Dex cytotoxicity in H929 and RPMI8226 cell lines. H929 and RPMI8226 cells were treated with indicated concentration of TAK-981 or Dex or both (combo) and cell viability was determined by Cell-Titer-Glo post-48h treatment. *Combination Index (CI) < 1 determined by CompuSym. (B) Knockdown of SAE2 and UBC9 enhances MM1R Dex sensitivity. MM1R cells were transfected with siRNA targeting SAE2 (SiSAE2) or UBC9 (SiUBC9), or non-targeting control (SiCtrl) for 48 h then treated with Dex (5 μM) for 24h. Apoptosis assay was performed using Annexin V-FITC/7-AAD. (C) Western blot of SAE2 and UBC9 level in s MM1R cells transfected with siRNA targeting SAE2 (SiSAE2) or UBC9 (SiUBC9), or non-targeting control (SiCtrl). GAPDH serves as loading control. (D) Western blot analysis shows SUMO-2,3 modifications, SAE2, and cleaved PARP (c-PARP) levels in RPMI8226 stable cell lines with SAE2 knockdown by two different shRNAs-shSAE2#1 and shSAE2#5. Dox (5 μg/mL) was added for 3 days to induce SAE2 knockdown (+Dox). GAPDH was used as loading controls. (E) Knockdown of SAE2 expression impairs MM cell growth. Cell viability of RPMI8226 stable cell lines. (F) Knockdown of SAE2 enhances Dex sensitivity in MM. RPMI8226 stable lines were treated with (+Dox) or without (-Dox) Dox (5 μg/mL) for 48 h followed by a series of concentration of Dex treatment for another 48 h. Cell viability was measured by cell-titer-glo assay and IC_{50} values of Dex in each group were calculated by GraphPad Prism 8. Estimated variation is indicated as SD. *P* values were derived using a two-tailed Student *t* test. *, p < 0.05, **, *P* < 0.01; ***, *P* < 0.001

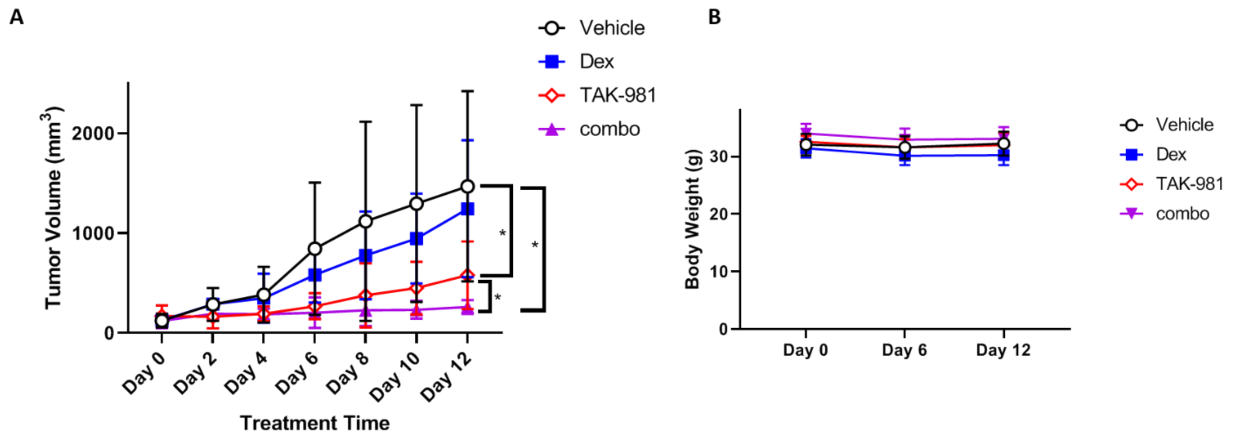

Supplementary Figure S3. TAK-981 treatment suppresses tumor growth in MM1R xenograft NSG mice model with synergistic effect in combination with Dex. NSG mice were xenografted by subcutaneously injection of MM1R cells ($4x10^6$ cells /mouse). Mice were treated with either vehicle, or Dex (3mg/kg), TAK-981 (10 mg/kg) or combination of both Dex and TAK-981(combo). All agents were treated twice weekly (BIW). (A) Tumor growth was evaluated by caliper measurement and represented as tumor volume (millimeters cubed). (B) Mice body weight was not affected by treatment in all groups. *P* values were derived using a two-tailed Student *t* test. *, $p < 0.05$.

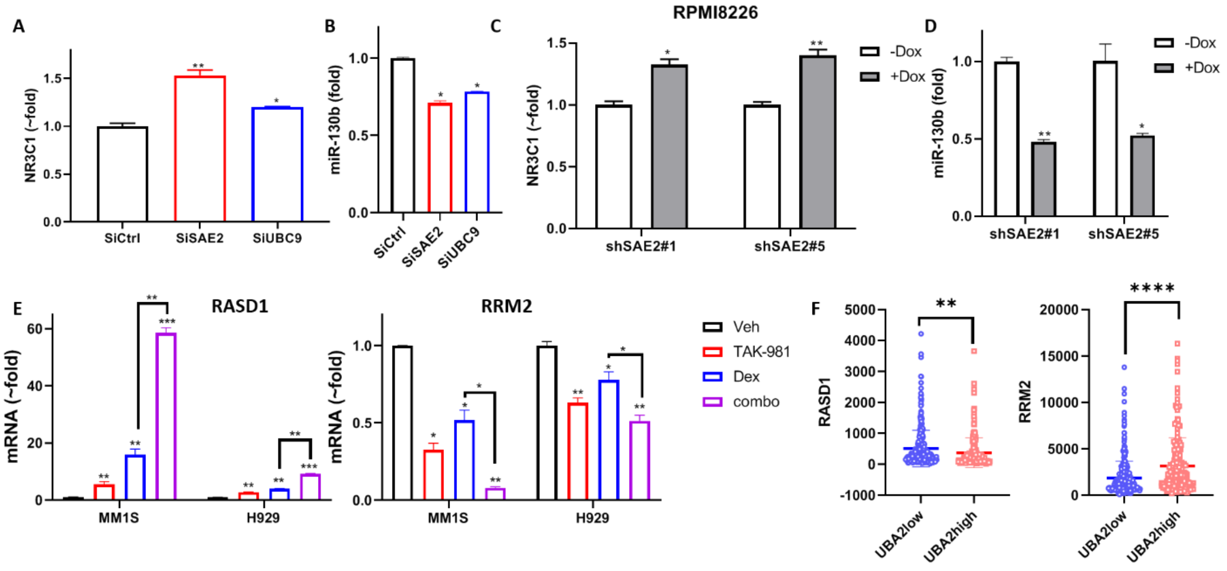

Supplementary Figure S4. SUMOylation inhibition upregulates GR expression with downregulation of miR-130b. (A) NR3C1 mRNA level and (B) miR-130b level were measured by qPCR in MM1S cells transfected with siRNA targeting SAE2 (SiSAE2) or UBC9 (SiUBC9), or non-targeting control (SiCtrl). (C) NR3C1 mRNA level and (D) miR-130b level were measured by qPCR in RPMI8226 stable lines with (+Dox) or without (-Dox) SAE2 knockdown. Estimated variation is indicated as SD. *P* values were derived using a two-tailed Student *t* test. *, p < 0.05; **, *P* < 0.01. (E) GR activated gene RASD1 and repressed gene RRM2 mRNA level measured by q-PCR using RNA extracted from MM1S and H929 cells treated with Vehicle (Veh), 0.1µM TAK-981 (TAK), 1 µM Dex (Dex), or 0.1 µM TAK-981 with 1 µM Dex (combo) for 48h. Estimated variation is indicated as SD. *P* values were derived using one-way ANOVA. (F) UBA2 level correlates with RASD1 and RRM2 expression in patient specimens. Analysis of cohort (GSE2658) of 559 MM patients (Supplemental Figure S1B). Patients with low SAE2 (UBA2; UBA2high group) showed higher RASD1 levels (left) and lower RRM2 level (right) than patients with high SAE2 (UBA2; UBA2low group). Data were analyzed using unpaired Student t tests: Data presented as mean±SD. *, p<0.05; **, p<0.01; ***, p<0.001, ****, p < 0.0001.

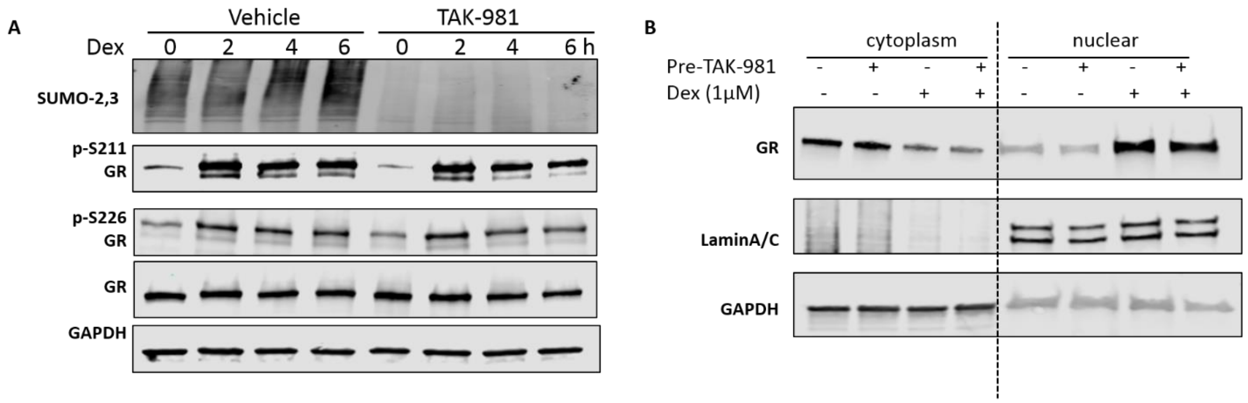

Supplementary Figure S5. TAK-981 doesn't affect GR phosphorylation and nuclear localization. (A) MM1S cell pre-treated with vehicle or TAK-981 (0.1μM) for 4 h were exposed to Dex (1 μM), cells were harvested at indicated time points and analyzed for phosphorylation of GR at Ser 211 (p-S211 GR) and Ser 226 (p-S226 GR) level, total GR level and global SUMOylation (SUMO-2,3) by western blot. (B) MM1S cell pre-treated with vehicle or TAK-981 (0.1μM) for 4 h were exposed to Dex (1 μM) for 2 h, cytoplasmatic and nuclear fraction were extracted and GR level was measured by western blot. Lamin A/C was used for nuclear fraction loading control and GAPDH for cytoplasmatic fraction loading control.

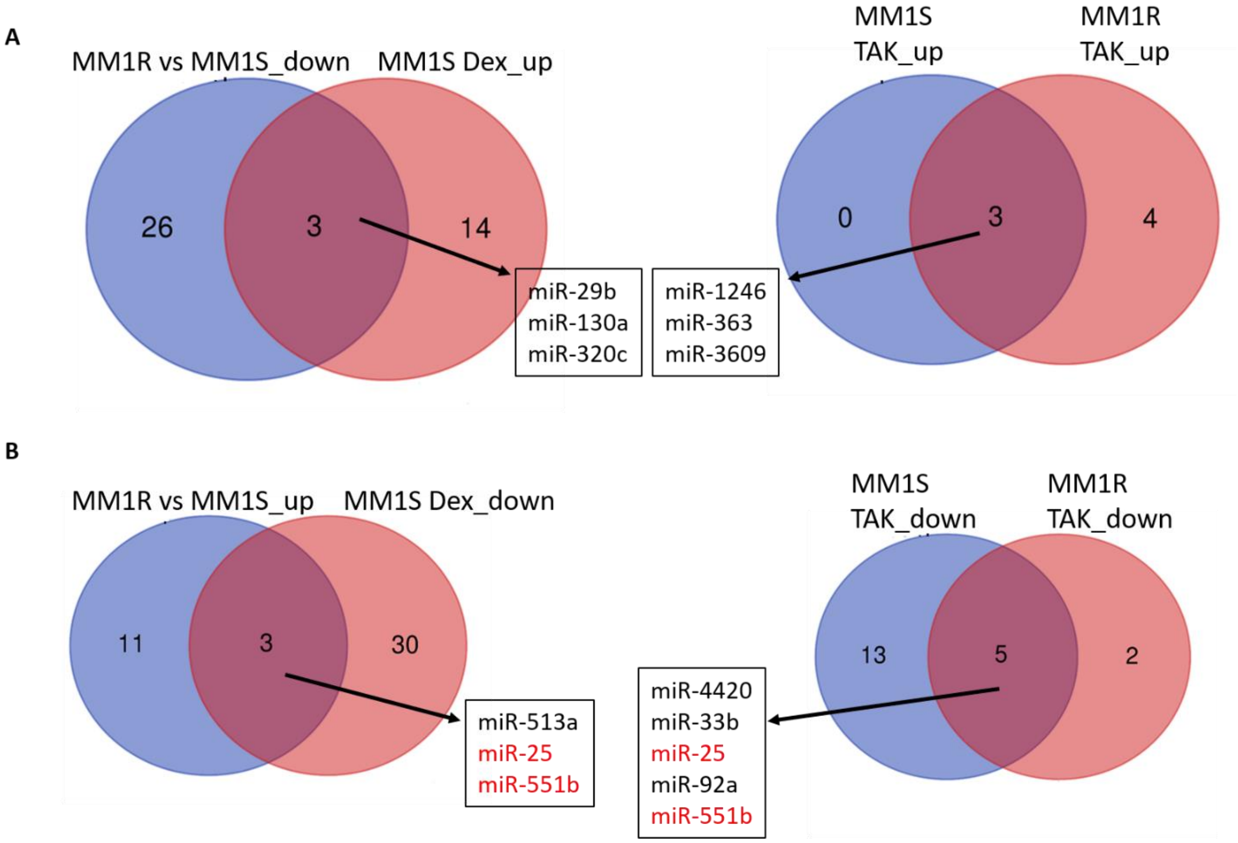

Supplementary Figure S6. Diagram presents the overlap between the statistically significant miRNAs from miR-seq analysis. miRs upregulated in MM1R compared to MM1S but downregulated upon Dex treatment or vice versa are considered mediating Dex sensitivity. These miRs are compared to miRs affected by TAK-981, the overlapping miRs are considered as involved in Dex sensitivity and regulated by SUMOylation. (A) Left: Overlapping miRs between downregulated miRs in MM1R compared to MM1S (MM1R vs MM1S_down) and upregulated miRs in MM1S by Dex treatment (MM1S Dex_up). Right: Overlapping miRs upregulated by TAK-981 treatment in both MM1S (MM1S TAK_up) and MM1R (MM1R TAK_up). (B) Left: Overlapping miRs between upregulated miRs in MM1R compared to MM1S (MM1R vs MM1S_up) and downregulated miRs in MM1S by Dex treatment (MM1S Dex_down). Right: Overlapping miRs downregulated by TAK-981 treatment in both MM1S (MM1S TAK_down) and MM1R (MM1R TAK_down). Two common miRNAs miR-551b and miR-25 are identified as potential miRs mediating Dex sensitivity and affected by SUMOylation inhibition.

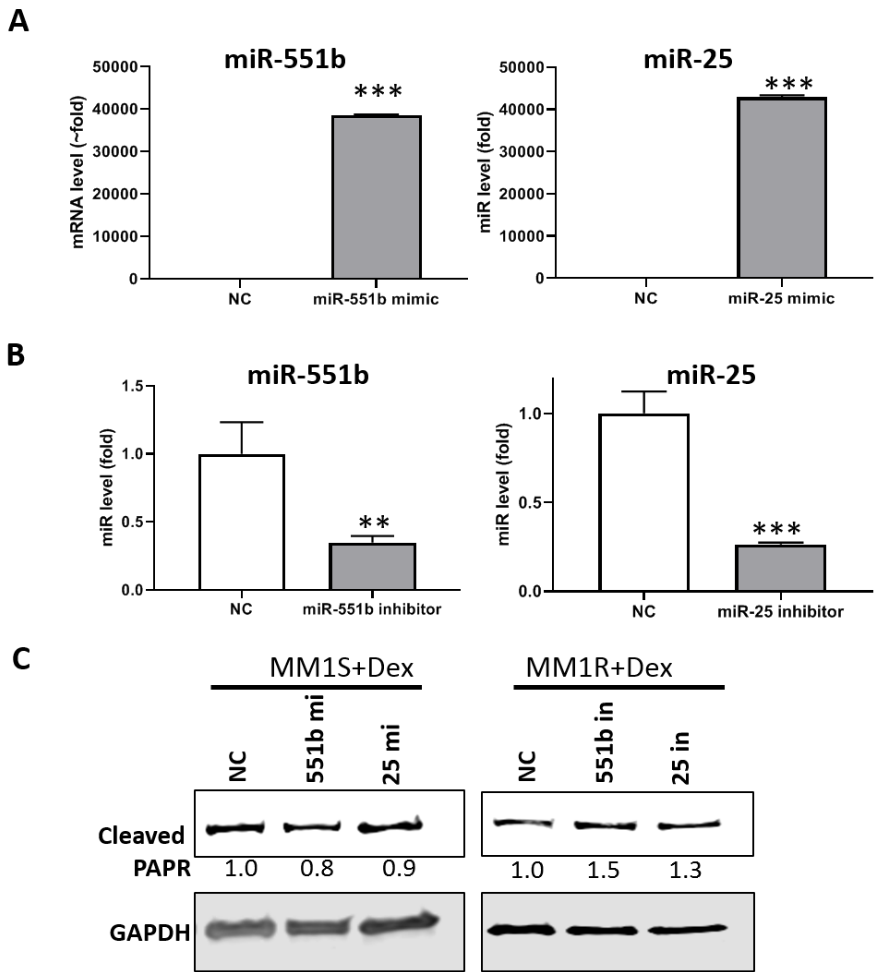

Supplementary Figure S7. MiR-551b and miR-25 level affect Dex sensitivity in MM.(A) miR-551b and miR-25 levels in MM1S cells transfected with miR-551b mimic, miR-25 mimic or non-targeting control siRNA (NC) by qPCR. (B) miR-551b and miR-25 levels in MM1R cells transfected with miR-551b inhibitor, miR-25 inhibitor or non-targeting control siRNA (NC) by qPCR. Estimated variation is indicated as SD. *P* values were derived using a two-tailed Student *t* test. **, $P < 0.01$; ***, $p < 0.001$. (C) cleaved PAPR (c-PARP) level was determined by western blot in MM1S cells transfected with microRNAs: miR-551b mimic, miR-25 mimic or non-targeting control (NC) with Dex (1µM) 24 h treatment and MM1R cells transfected with anti-miRs: miR-551b inhibitor, miR-25 inhibitor or non-targeting control (NC) with Dex (5µM) 24 h treatment. GAPDH was used as loading control.

A

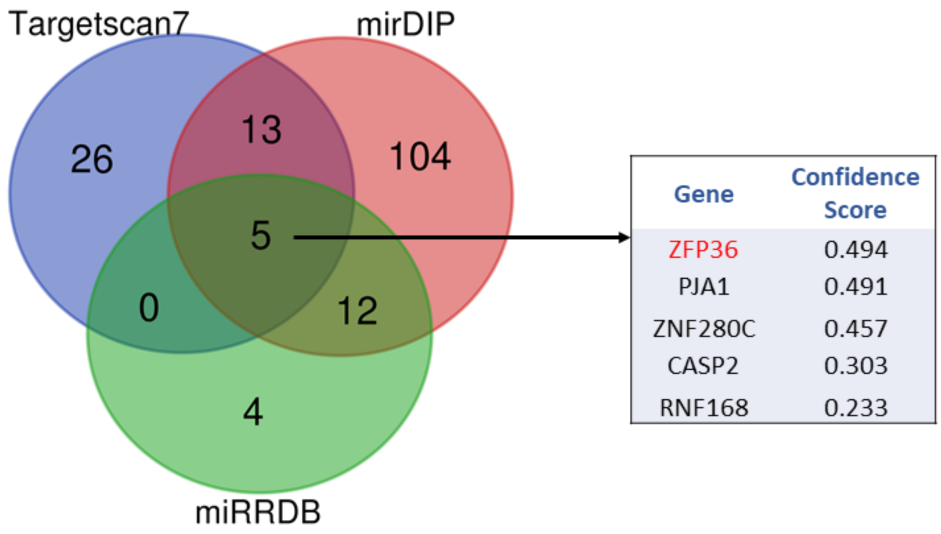

B

ZFP36 3'UTR 5'-..GTATTTATATATAT**TGGGTCG**T.. -3'

miR-551b 3'-GACUUUGGUUCAU**ACCCAGC**G-5'

Supplementary Figure S8. Analysis of possible miR-551b target gene and binding sites. (A) Venn diagram showing potential target genes of miR-551b in 3 databases. Among the 5 genes overlapped in the 3 databases, ZFP36 ranged the highest confidence as a target of miR-551b. Confidence score measured by an algorithm from mirDIP, higher scores indicate more confidence of the prediction. (B) miR-551b binding site within ZFP36 3'UTR.

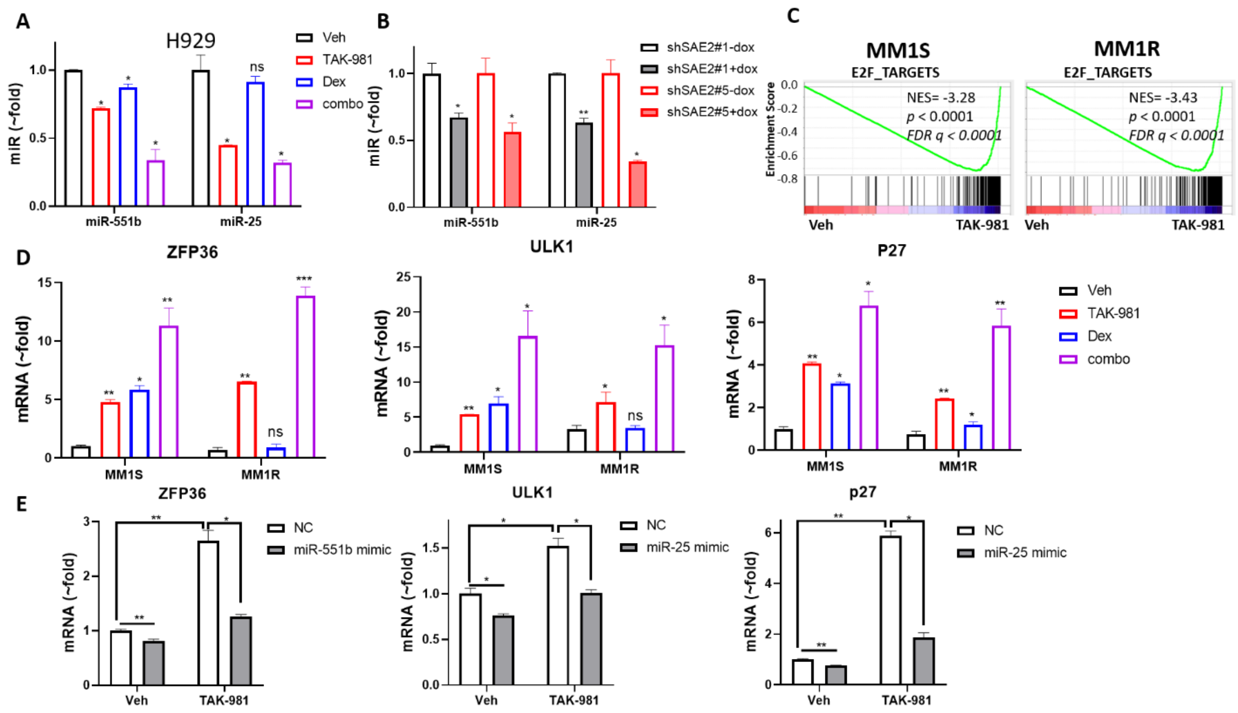

Supplementary Figure S9. SUMOylation inhibition decreases miR-551b and miR-25 level. (A) miR-551b and miR-25 levels were decreased by TAK-981 treatment in H929 cells. H929 cells were treated with Vehicle (Veh), 0.1µM TAK-981 (TAK), 1 µM Dex (Dex), or 0.1 µM TAK-981 with 1 µM Dex (combo) for 48h. miR level was measured by Taqman q-PCR. (B) Knockdown of SAE2 reduced expression of miR-551b and miR-25 in RPMI8226 cells. miR-551b and miR-25 levels were measured by qPCR in RPMI8226 stable lines with (+Dox) or without (-Dox) SAE2 knockdown. (C) GSEA from genome-wide mRNA-seq shows E2F targets were down-regulated upon TAK-981 treatment in both MM1S and MM1R cells. (D) ZFP36, ULK1 and p27 mRNA levels in MM1S and MM1R cells treated with Vehicle (Veh), 0.1µM TAK-981 (TAK), 1 µM Dex (Dex), or 0.1 µM TAK-981 with 1 µM Dex (combo) for 48h. (E) ZFP36, ULK1 and p27 mRNA levels in MM1S cells transfected with miR-551b mimic, miR-25 mimic or non-targeting control siRNA (NC) then treated with Vehicle (Veh) or 0.1 Mm TAK-981 (TAK-981) for 48h.Estimated variation is indicated as SD. *P* values were derived using a two-tailed Student *t* test. Ns, not significant; *, p < 0.05; **, *P* < 0.01.

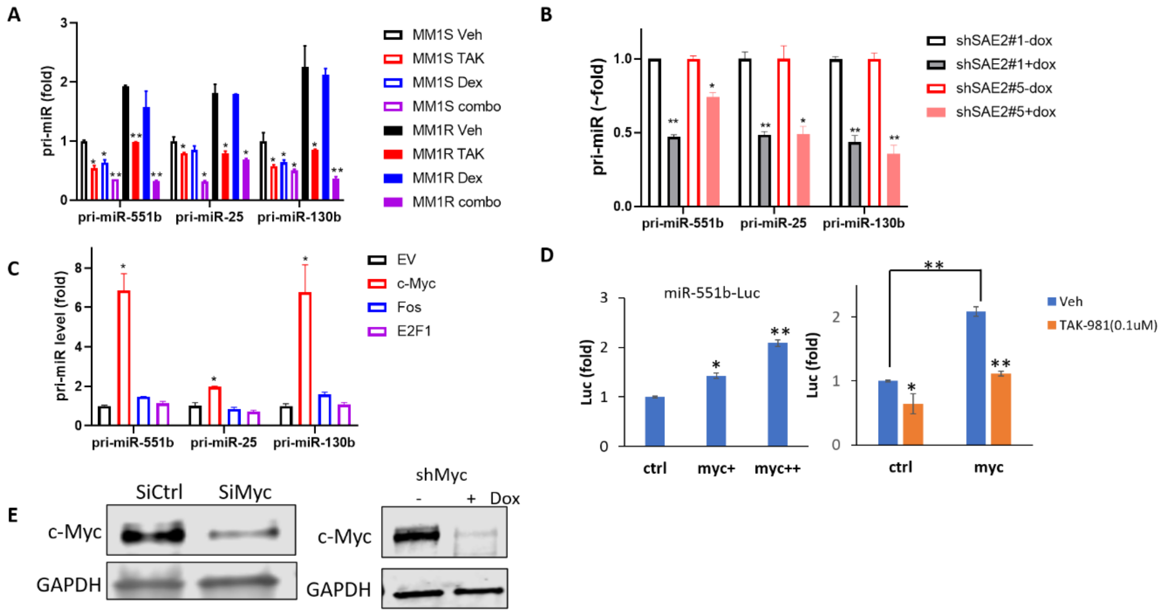

Supplementary Figure S10. SUMOylation inhibition decreases the expression miR-551b, miR-25 and miR-130b via c-Myc. (A) Pri-miR levels of pri-miR-551b, pri-miR-25 and pri-miR-130b were decreased by TAK-981 treatment in MM1S and MM1R cells. MM1S and MM1R cells were treated with Vehicle (Veh), 0.1µM TAK-981 (TAK), 1 µM Dex (Dex), or 0.1 µM TAK-981 with 1 µM Dex (combo) for 48h. pri-miR level was measured by Taqman q-PCR. (B) Knockdown of SAE2 decreased Pri-miR levels of pri-miR-551b, pri-miR-25 and pri-miR-130b. Pri-miR levels were measured by qPCR in RPMI8226 stable lines with (+Dox) or without (-Dox) SAE2 knockdown. (C) Pri-miR levels of pri-miR-551b, pri-miR-25 and pri-miR-130b were significantly enhanced by c-Myc expression, but not by Fos or E2F1 expression. MM1S cells were transfected with with empty vector (EV) or c-Myc, Fos, or E2F1 expression plasmids. Pri-miR levels were measured by Taqman qPCR after 48 h. (D) miR-551b promoter transcriptional activity was significantly enhanced by c-Myc expression and suppressed by TAK-981 treatment. 293T cells were transfected with empty vector (Ctrl) or c-Myc expression plasmids at two doses, 100 ng/well (+) and 200 ng/well (++), together with miR-551b promoter luciferase reporter and Renilla plasmids. After 24 h, cells were treated with 0.1µM TAK-981 or vehicle for 16 h then dual-luciferase activity was measured. Estimated variation is indicated as SD. *P* values were derived using a two-tailed Student *t* test. *, $p < 0.05$; **, $P < 0.01$. (E) Western blot of c-Myc level in MM1S cells transfected siRNA targeting c-Myc (siMyc) or control siRNA (SiCtrl) (main figure 6B) and RPMI8226 stable line with (+Dox) or without (-Dox) c-Myc knockdown (main figure 6C).

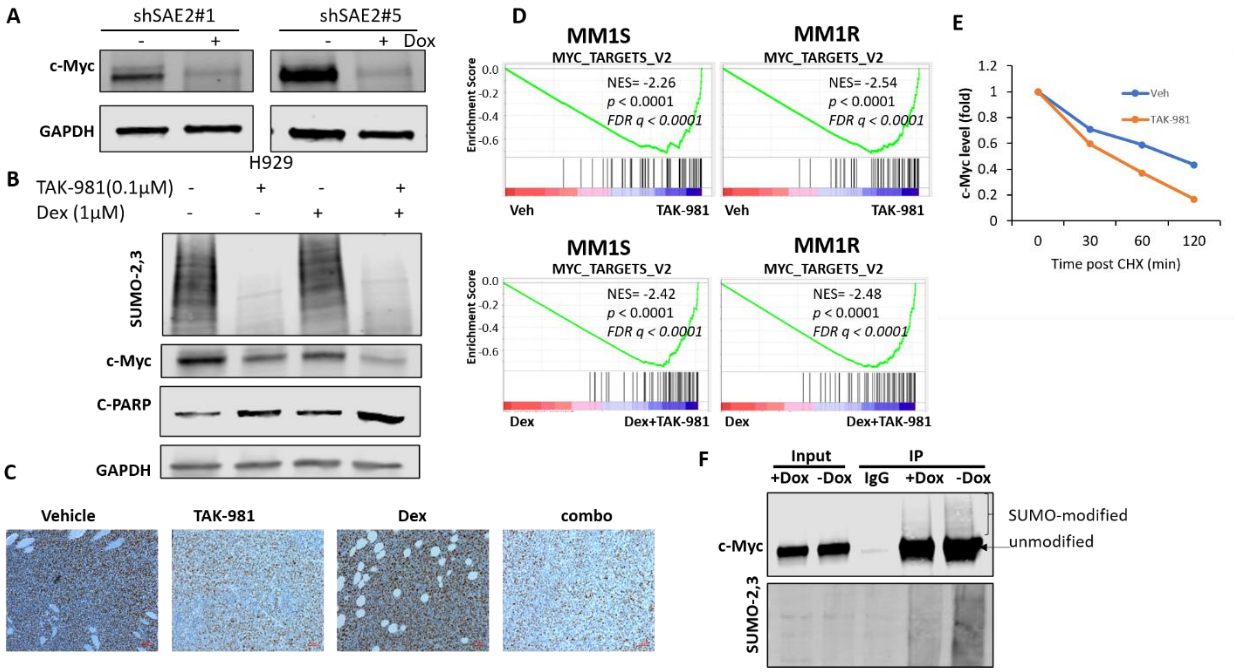

Supplementary Figure S11. SUMOylation inhibition decreased c-Myc protein level. (A) western blot of c-Myc level in RPMI8226 two stable lines with (+Dox) or without (-Dox) SAE2 knockdown. (B) TAK-981 treatment decreases c-myc levels and further decreases c-Myc in combination with Dex in H929 cells. H929 cells were treated with TAK-981 or Dex or both for 48 h, c-myc and cleaved PAPR (c-PARP) levels were determined by western blot. SUMO-2,3 was blotted to determine global SUMOylation and GAPDH was served as loading control. (C) IHC staining of c-Myc expression in xenograft tumor tissues. Red scale bar: 50 µm. (D) GSEA analysis of RNA-seq data shows TAK-981 treatment inhibits c-Myc target gene sets (MYC_TARGETS_V2) in MM1S and MM1R cells. (E) Quantification plot of c-Myc level in TAK-981 or vehicle treated MM1R cells. (main figure 7D). (F) SUMO modification of c-Myc was reduced by SAE2 knockdown. Immunoprecipitation assay was performed using c-Myc antibody in RPMI8226 cells with (+Dox) or without (−Dox) SAE2 knockdown and c-Myc and SUMO2,3 were blotted. In order to rule out the decrease effect of c-Myc after SAE2 knockdown, 2 fold of protein amount from cell lysate in with (+Dox) than without (−Dox) SAE2 knockdown was used for IP to match the amount of unmodified c-Myc protein level.

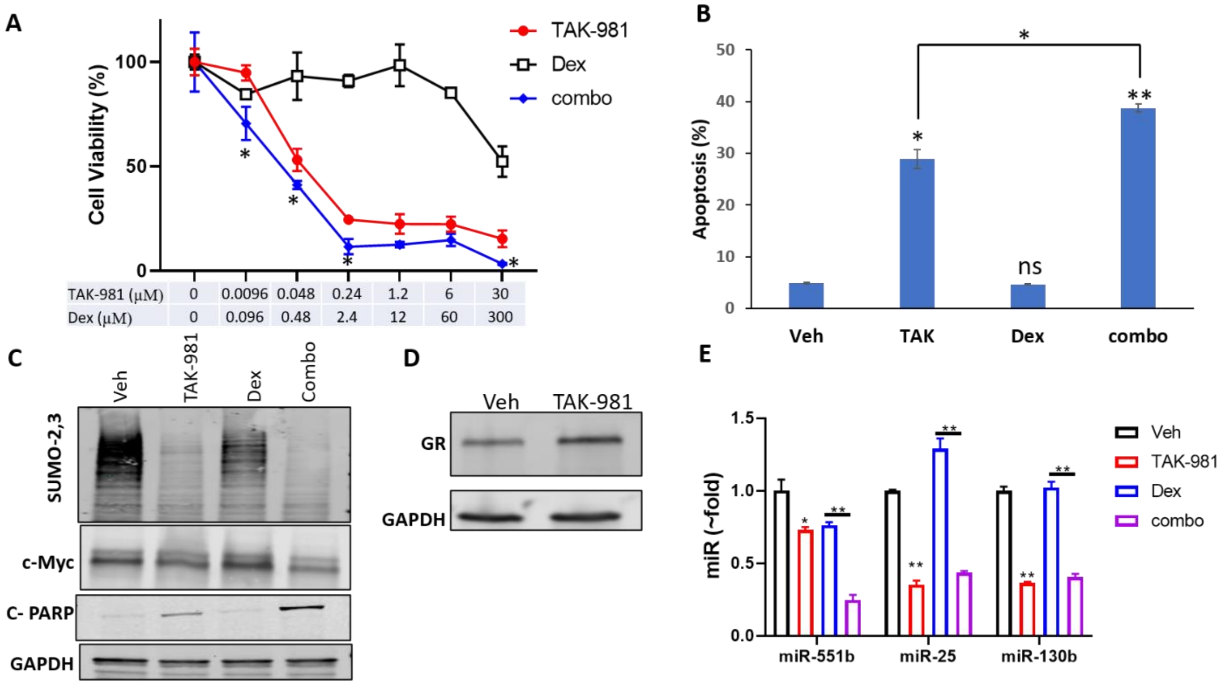

Supplementary Figure S12. TAK-981 synergizes with Dex in decreasing cell viability in Jurkat via c-Myc mediated miR and GR regulation. (A) TAK-981 synergizes with Dex cytotoxicity in Jurkat cells. Jurkat cells were treated with indicated concentration of TAK-981 or Dex or both (combo) and cell viability was determined by Cell-Titer-Glo post-48h treatment. *Combination Index (CI) < 1 determined by CompuSym. (B) TAK-981 enhances cytotoxicity of Dex in Jurkat cells. Jurkat cells were treated with Vehicle (Veh), 0.1µM TAK-981 (TAK), 1 µM Dex (Dex), or 0.1 µM TAK-981 with 1 µM Dex (combo). Apoptosis was measured by flow cytometry using Annexin V/7-AAD staining. (C) TAK-981 treatment synergizes with Dex in inducing apoptosis marker cleaved PARP expression and decreasing c-myc level in Jurkat cells. Jurkat cells were treated with TAK-981 or Dex or both for 48 h, c-Myc and cleaved PAPR (c-PARP) levels were determined by western blot. SUMO-2,3 was blotted to determine global SUMOylation and GAPDH was served as loading control. (D) TAK-981 treatment induces GR protein level by western blot. (E) TAK-981 treatment decreases expression level of miR-551b, miR-25 and miR-130b. Estimated variation is indicated as SD. *P* values were derived using a two-tailed Student *t* test. *, $p < 0.05$; **, $P < 0.01$.
